# A Virtual Breakthrough Series Collaborative for Missed Test Results: A Stepped-Wedge Cluster-Randomized Clinical Trial

**DOI:** 10.1001/jamanetworkopen.2024.40269

**Published:** 2024-10-30

**Authors:** Lisa Zubkoff, Andrew J. Zimolzak, Ashley N. D. Meyer, Jennifer Sloane, Umber Shahid, Traber Giardina, Sahar A. Memon, Taylor M. Scott, Daniel R. Murphy, Hardeep Singh

**Affiliations:** 1 Birmingham/Atlanta Geriatric Research Education and Clinical Center, Birmingham VA Healthcare System, Birmingham, Alabama; 2 Division of Preventive Medicine, University of Alabama at Birmingham, Birmingham; 3 Center for Innovations in Quality, Effectiveness and Safety, Michael E. DeBakey Veterans Affairs Medical Center, Houston, Texas; 4 Department of Medicine, Baylor College of Medicine, Houston, Texas

## Abstract

**Importance:**

Missed test results, defined as test results not followed up within an appropriate time frame, are common and lead to delays in diagnosis and treatment.

**Objective:**

To evaluate the effect of a quality improvement collaborative, the Virtual Breakthrough Series (VBTS), on the follow-up rate of 2 types of test results prone to being missed: chest imaging suspicious for lung cancer and laboratory findings suggestive of colorectal cancer.

**Design, Setting, and Participants:**

This stepped-wedge cluster-randomized clinical trial was conducted between February 2020 and March 2022 at 12 Department of Veterans Affairs (VA) medical centers, with a predefined 3-cohort roll-out. Each cohort was exposed to 3 phases: preintervention, action, and continuous improvement. Follow-up ranged from 0 to 12 months, depending on cohort. Teams at each site were led by a project leader and included diverse interdisciplinary representation, with a mix of clinical and technical experts, senior leaders, nursing champions, and other interdisciplinary team members. Analysis was conducted per protocol, and data were analyzed from April 2022 to March 2024.

**Intervention:**

All teams participated in a VBTS, which included instruction on reducing rates of missed test results at their site.

**Main Outcomes and Measures:**

The primary outcome was changes in the percentage of abnormal test result follow-up, comparing the preintervention phase with the action phase. Secondary outcomes were effects across cohorts and the intervention’s effect on sites with the highest and lowest preintervention follow-up rates. Previously validated electronic algorithms measured abnormal imaging and laboratory test result follow-up rates.

**Results:**

A total of 11 teams completed the VBTS and implemented 47 (mean, 4 per team; range, 3-8 per team; mode, 3 per team) unique interventions to improve missed test results. A total of 40 027 colorectal cancer–related tests were performed, with 5130 abnormal results, of which 1286 results were flagged by the electronic trigger (e-trigger) algorithm as being missed. For lung cancer–related studies, 376 765 tests were performed, with 7314 abnormal results and 2436 flagged by the e-trigger as being missed. There was no significant difference in the percentage of abnormal test results followed up by study phase, consistent across all 3 cohorts. The estimated mean difference between the preintervention and action phases was −0.78 (95% CI, −6.88 to 5.31) percentage points for the colorectal e-trigger and 0.36 (95% CI, −5.19 to 5.9) percentage points for the lung e-trigger. However, there was a significant effect of the intervention by site, with the site with the lowest follow-up rate at baseline increasing its follow-up rate from 27.8% in the preintervention phase to 55.6% in the action phase.

**Conclusions and Relevance:**

In this cluster-randomized clinical trial of the VBTS intervention, there was no improvement in the percentage of test results receiving follow-up. However, the VBTS may offer benefits for sites with low baseline performance.

**Trial Registration:**

ClinicalTrials.gov Identifier: NCT04166240

## Key Points

**Question:**

To what extent does participation in a quality improvement collaborative, the Virtual Breakthrough Series (VBTS), reduce the rate of missed abnormal test results?

**Findings:**

In this stepped-wedge cluster-randomized clinical trial conducted at 12 Department of Veterans Affairs medical centers randomized to 3 implementation timelines, the overall percentage of missed abnormal test results did not change after the introduction of the intervention. Findings were consistent across all 3 cohorts.

**Meaning:**

This stepped-wedge cluster-randomized clinical trial found that the VBTS intervention did not impact rates of missed abnormal test results in need of follow-up.

## Teaser

This stepped-wedge cluster-randomized clinical trial evaluates the effect of a quality improvement collaborative, the Virtual Breakthrough Series, on the follow-up rate of 2 types of test results prone to being missed: chest imaging suspicious for malignant neoplasms and laboratory findings suggestive of colorectal cancer.

## Introduction

Timely follow-up of test results is a common safety concern.^1,2,3^ Several studies have shown how missed abnormal test results (ie, results outside reference ranges or suggestive of an underlying health problem or pathology not followed up within an appropriate time frame) can lead to delays in diagnosis and treatment of cancers.^4,5,6,7,8^ Systematic reviews have described a variety of electronic interventions to improve test result follow-up, including discharge summary templates, email alerts for pending test results, automated notifications, results acknowledgment systems, computerized clinician order entry, and clinical information systems to facilitate closed-loop communication.^9,10,11^ Quality improvement approaches have also shown promise in reducing ambulatory malpractice risk related to missed test results.^12^ Electronic methods, such as electronic triggers (e-triggers), can identify patients who lack timely follow-up of their test results and help notify clinicians.^8^ While health care organizations are often aware of such solutions, implementation has been inconsistent, resulting in an implementation gap.^13^

The Breakthrough Series, developed by the Institute for Healthcare Improvement (IHI), is a face-to-face quality improvement collaborative model that uses planned change theory.^14^ It is designed to help organizations close the gap between what practitioners and systems know and what they do, using a short-term (approximately 6 months) learning experience that brings together teams from hospitals to improve care in a specific area. The model includes 4 key elements (aims, measurement, implementing changes, and testing small cycles of change^14^) and has gained traction during the past 15 years.^15,16,17,18^

In 2006, IHI described a Virtual Breakthrough Series (VBTS),^19^ and in 2011, the US Department of Veterans Affairs (VA) National Center for Patient Safety adapted it for virtual use. A VBTS is a remote collaborative designed to close implementation gaps and improve quality through educating and coaching.^19,20^ Prior VBTSs have improved outcomes in postoperative complications,^21^ catheter-associated urinary tract infections,^22^ falls,^23,24^ and pressure ulcers.^25,26^ Despite their prevalence,^1,2,3^ potential for harm, and presence of an implementation gap,^13^ VBTS methods have not been applied to reduce missed test results, to our knowledge.

In US ambulatory care, care fragmentation and inadequate data systems make tracking patients’ longitudinal care difficult, hindering our ability to identify, assess, and reduce missed test results. The VA runs the most extensive integrated health care system in the US, with a longitudinal electronic health record (EHR) data repository that spans the care journey and allows the detection of missed test results and intervention effects. Thus, we conducted a stepped-wedge cluster-randomized clinical trial (SW-CRCT) in the VA^27^ to evaluate the effect of the VBTS on the rate of missed0020test results suggestive of colorectal or lung cancer.

## Methods

This SW-CRCT was approved by the Baylor College of Medicine institutional review board and VA Research and Development. We were granted a waiver of consent because it was not feasible to obtain consent for medical record reviews from the large number of patients included in the study. This study is reported following the Reporting of Stepped Wedge Cluster Randomised Trials: Extension of the CONSORT 2010 Statement (RS-WRT CONSORT) reporting guideline. The trial protocol and statistical analysis plan are provided in Supplement 1.

### Trial Design

**Figure 1.**
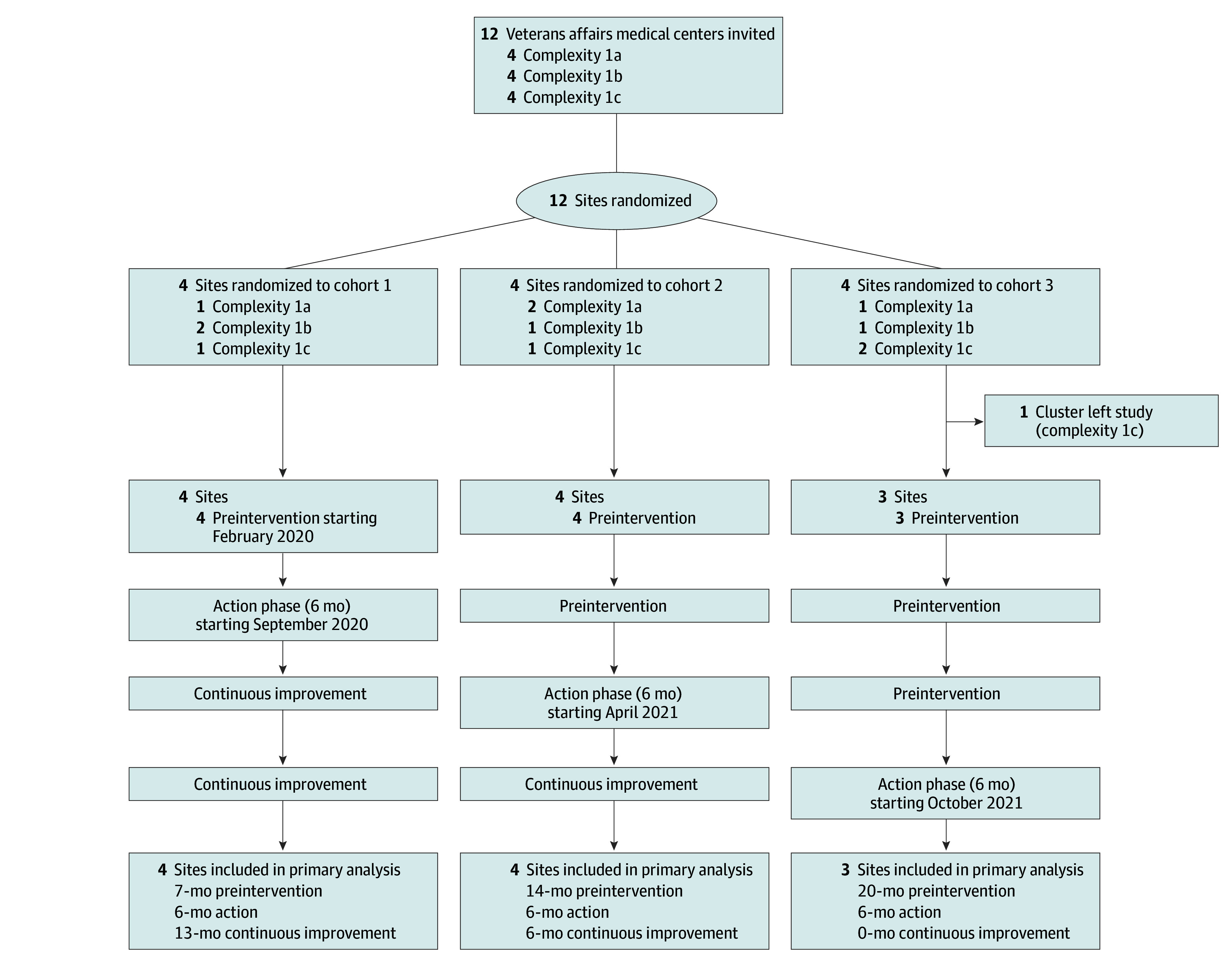
Flow of Patients Through the Study For all sites, the final 3 months of the preintervention phase allowed sites to review materials and meet with the study team. Action phase was 6 months and comprised monthly education sessions, implementation coaching, and plan-do-study-act cycles. Continuous improvement included coaching on request from any site.

We conducted a 26-month SW-CRCT at 12 VA Medical Centers from February 2020 to April 2022 (Figure 1). We evaluated the effect of a VBTS on the rate of follow-up of test results that warrant follow-up diagnostic evaluation for lung or colorectal cancer. For lung cancer, tests included chest radiograph or computed tomography studies coded by the radiologist as suspicious for malignant neoplasms. For colorectal cancer, tests included positive fecal occult blood test results, positive fecal immunochemical test results, or blood test results suggestive of iron deficiency anemia. The study was initially conceived as beginning in October 2019, with intervention beginning January 2020, but the COVID-19 pandemic interrupted the intervention, so it was restarted in September 2020.

### Trial Sites

We recruited teams from VA medical centers nationwide (hereafter, *sites*) by promoting the study at meetings and conferences and emailing site leadership. We used purposive recruitment to ensure sites represented a range of complexity and geography. The VA uses a standardized Facility Complexity Model (eTable 1 in Supplement 2) that assigns 5 levels of complexity to sites based on patient volume, patient risk, clinical services complexity, and extent of research and trainee programs.^28^ Patients were included if they had any of the following at the site: chest radiography or computed tomography, fecal occult blood tests, fecal immunochemical tests, or blood counts suggestive of iron deficiency anemia. The study team met with interested sites to discuss goals, duration, and time commitment.

### Randomization

Each site was randomized to 1 of 3 cohorts while balancing facility complexity among cohorts and limiting cohorts to 4 sites (Figure 1). Sites were allowed to change personnel if staffing dictated. Sites that entered the preintervention phase remained in analysis regardless of activity or performance.

### Trial Intervention: VBTS

The trial intervention had 4 components: contact with VBTS faculty, change package, workbook, and e-trigger algorithms. VBTS faculty included VA clinicians, health services and safety researchers, informaticists, and implementation scientists.

To guide the VBTS, we developed a change package, defined as “a catalogue of strategies, change concepts, and action steps that guide participants in their improvement efforts.”^29^ The change package gave sites a menu of interventions to reduce missed test results and strategies for change. Change package development involved semistructured interviews with multidisciplinary VA personnel and thematic analysis to determine factors contributing to missed test results.^13^ The change package was organized into 3 evidence-based primary drivers or goals: enhancing patient engagement with test results, improving situational awareness among all clinicians and care teams, and implementing processes to close the loop on test result reporting and follow-up. Each primary driver was associated with several secondary drivers and action steps that teams could implement. Definitions and evidence for secondary drivers are included in the change package (eFigure 1 in Supplement 2).

We created a workbook to orient participants and to provide them with technical resources. It included a collaborative charter (mission, goal, timeline, expectations) and an overview of VBTS phases. Lastly, we adapted previously developed Structured Query Language e-trigger algorithms for sites to identify patients with potentially missed test results and track follow-up rates.^30^ E-triggers mine EHR data to identify patterns suggesting delays, such as missed follow-up opportunities after abnormal test results. E-trigger logic, criteria for abnormal test results, and follow-up time-frames are described elsewhere.^31^ This study did not conduct medical record audits to confirm presence or absence of follow-up; hence, results were considered potentially missed. Patient records flagged by the e-trigger are highly likely to have a missed opportunity for follow-up of an abnormal test result.^6,7,32,33^ Using these materials, VBTS faculty provided coaching on interventions to reduce missed test results, process improvement strategies, and how to implement e-triggers.^30^

### Study Phases

We designed this study as a SW-CRCT with 3 cohorts to allow sufficient coaching and contact with sites. Each cohort participated in 3 consecutive phases: preintervention, action, and continuous improvement. Continuous improvement follow-up varied: 12 months for cohort 1, 6 months for cohort 2, and 0 months for cohort 3. Outcome data from these phases spanned 26 months for each cohort (Figure 1).

The preintervention phase varied in duration (Figure 1), with the final 3 months of each preintervention phase consisting of VBTS preparation. Each site was asked to form a multidisciplinary team to lead changes, review baseline data, investigate current processes, develop aims, and complete an initial plan. Teams were expected to include a project leader, clinical expert, senior leader, nursing champion, and other interdisciplinary team members as appropriate (eg, systems redesign, quality improvement, radiology and laboratory, and informatics and data personnel). In the final 3 months of the preintervention phase, the study team held monthly calls with all sites in the cohort, covering topics such as VBTS goals, roles and expectations of team members, role of VBTS faculty, data collection, and methods for examining current care processes. Sites were provided the workbook, change package, and e-trigger algorithms. The study team ensured each site could run the e-triggers, but sites did not review or act on e-trigger numbers until the action phase.

During the action phase (6 months), teams submitted monthly reports on activities and their impact on test result follow-up, as measured by aggregate e-trigger data (count of trigger-positive tests and total tests with abnormal results). In monthly group calls, teams were taught evidence-based changes, plan-do-study-act cycles,^18^ and possible interventions. Teams reported impacts during these calls and discussed successes and challenges. We provided coaching on the monthly calls and in writing after reviewing monthly reports. Coaches were experts in missed test results, data, and quality improvement. At the end of the action phase, teams presented a work summary.

In the continuous improvement phase, teams did not submit monthly reports or have scheduled calls with the study team, but coaches were available as needed. Continuous improvement varied in duration (Figure 1).

### Outcomes

Final reports submitted by the teams at the end of the action phase were used to quantify the number and type of interventions implemented. Interventions were coded by consensus by 2 authors (A.J.Z. and L.Z.). If agreement could not be reached, a third team member was consulted.

The primary outcome was the change in the follow-up rate of abnormal test results suggestive of lung or colorectal cancer from the preintervention phase to the action phase. This outcome was determined using standard data retrieval techniques for e-triggers by the central research team. We hypothesized there would be higher test result follow-up during the intervention period than the preintervention period. As a secondary outcome, we assessed the change in the follow-up rate of abnormal test results suggestive of lung or colorectal cancer from the preintervention phase to the continuous improvement phase.

Because the intervention for cohort 1 was affected by the start of the COVID-19 pandemic, we conducted an exploratory analysis to test for differences in changes of follow-up rates from cohort to cohort. In another exploratory analysis, we hypothesized that high-performing sites before the intervention may experience a ceiling effect and would be less likely to experience a significant effect of the intervention. Therefore, we compared the effect size between the 2 sites with the maximum and minimum baseline performance. Baseline was defined as the mean monthly follow-up rate in the preintervention phase for each site and test type (lung cancer evaluation e-trigger and colorectal cancer evaluation e-trigger).

### Statistical Analysis

Power analyses of primary outcomes were conducted per protocol using the method by Hemming and Girling^34,35^ for determining power for SW-CRCTs comparing performance in preintervention vs action phases (assuming 2-tailed α = .05; intraclass correlation = 0.10). Assuming 12 sites for feasibility, a mean improvement of follow-up of abnormal test results from 56% in the preintervention phase to 67% in the action phase gives 82% power to detect a significant difference in colorectal cancer evaluation–related e-trigger performance.^6^ Similarly, a mean improvement of follow-up of abnormal test results from 61% in the preintervention phase to 73% in the action phase gives 89% power to detect a significant difference in lung cancer evaluation–related e-trigger performance.^32^

We compared e-trigger rates among the 3 phases using linear mixed-effects models^36^ to compute estimated mean differences in follow-up with 95% CIs. Models compared preintervention vs action phases (primary outcome) and preintervention vs continuous improvement phases (secondary outcome) by including study phase as a fixed effect, study month (number 1-26) as a fixed effect to account for calendar time, and individual site as a random effect.

For the exploratory analysis, we used analysis of variance to model the interaction of cohort and study phase and their effect on the percentage of test results followed up. All tests were 2-tailed, and *P* < .05 was considered significant. Analyses were performed using Stata version 11 (StataCorp) and R version 4.2.1 (R Project for Statistical Computing) with the lme4 package.^37^ Data were analyzed from April 2022 to March 2024.

## Results

Of 12 sites initially enrolled, 11 sites completed the VBTS (Figure 1). One site in cohort 3 withdrew before the preintervention phase because of COVID-19–related workforce changes. A total of 40 027 colorectal cancer–related tests were performed, with 5130 abnormal test results and 1286 e-trigger–positive results indicating missed tests. For lung cancer–related studies, 376 765 tests were performed, with 7314 abnormal rest results and 2436 e-trigger–positive results. For the colorectal cancer e-triggers, sites’ baseline performance (mean follow-up rate in pre-intervention) ranged from 30.6% to 91.8% (median, 74.1%). For the lung cancer e-triggers, baseline performance ranged from 64.4% to 78.8% (median, 68.3%).

### Uptake of the Trial Intervention

Teams implemented 47 unique interventions (Table 1; eTable 2 in Supplement 2), with a mean of 4 per team, a range of 3 to 8 per team, and a mode of 3 per team. The most frequently implemented interventions were increasing patients’ access to test results via portals (10 sites [91%]), preventing clinician EHR notification fatigue using filtering and decreasing low-value notifications (6 sites [55%]), and monitoring for breakdowns in test results review and communication (5 sites [45%]). Teams also implemented novel interventions not directly mentioned in the change package but available through change package links and resources: updating policies and procedures (eg, steps to ensure follow-up by backup clinicians who receive test result notifications [2 sites]), training clinicians on test result follow-up strategies (1 site), quality improvement tools (eg, process mapping of test result notification and follow-up [2 sites]), procedures to more effectively label results as abnormal (2 sites), pay for performance to reward timely follow-up (1 site), and facilitating patient transportation to reduce no-shows in gastroenterology (1 site).

**Table 1.** Coding of Interventions Implemented by Sites^a^

Primary and secondary drivers	Sites, No. (n = 11)
Patient engagement	
Increase access to test results	10
Educate patients on expectations of the test result and follow-up process	3
Increase patient comprehension of test results	2
Situational awareness	
Prevent clinician EHR notification fatigue	6
Ensure effective teamwork in the management of test results	4
Create a support structure to facilitate test results review and follow-up	1
Facilitate time for clinicians to review and act on test results	0
Close the loop	
Ensure clinicians and staff have the necessary contact information for fail-safe communication	2
Monitor for breakdowns in test results review and communication	5
Use standardization and failsafe processes and policies for communicating test results in need of follow-up	1
Use EHR features to support closing the loop on test results	4
Novel drivers	
Update policies and procedures	2
Education of clinicians (other than notification fatigue)	1
Demonstrated use of quality improvement tools (eg, process mapping)	2
Procedure to more effectively label a result needing follow-up as such	2
Pay for performance for primary care physicians	1
Encourage gastroenterology clinic to discuss transportation, to reduce no-show rate	1
Total	47

Abbreviation: EHR, electronic health record.

^a^ A detailed description of all interventions implemented across sites is provided in eTable 2 in Supplement 2.

### Primary and Secondary Outcomes

Monthly e-trigger data were available for all 11 participating teams. This is a null study, as there were no detectable differences in the percentage of abnormal test results followed up by study phase in any of the cohorts (Table 2). The estimated mean difference between the preintervention and action phases (primary outcome) was −0.78 (95% CI, −6.88 to 5.31) percentage points for the colorectal cancer e-trigger and 0.36 (95% CI, −5.19 to 5.90) percentage points for the lung cancer e-trigger. Similarly, the estimated mean difference between the preintervention and continuous improvement phases was −0.02 (95% CI, −8.59 to 8.55) percentage points for the colorectal cancer e-trigger and 0.89 (95% CI, −6.85 to 8.63) percentage points for the lung cancer e-trigger (eFigure 2 and eFigure 3 in Supplement 2).

**Table 2.** Baseline and Outcome Characteristics by Study Phase

Characteristic	No.		
	Preintervention phase	Action phase	Continuous improvement phase
**Colorectal cancer**			
Facilities	11	11	8
Facility-months	144	66	76
Total tests	20 373	10 062	9592
Abnormal test results	2718	1187	1225
Trigger-positive test results	650	307	329
Patients with tests	18 461	9385	9399
Patients with abnormal test results	2610	1156	1201
Patients with trigger-positive test results	634	300	328
Site follow-up rate, mean % (percentage-point SD)	76.0 (19.3)	75.4 (15.5)	76.2 (20.0)
Difference vs preintervention, mean (95% CI), percentage points	NA	−0.78 (−6.88 to 5.31)^a^	−0.02 (−8.59 to 8.55)^b^
*P* value	NA	.80	>.99
**Lung cancer**			
Facilities	10	10	8
Facility-months	124	60	76
Total tests	170 596	89 866	116 303
Abnormal test results	2888	1553	2873
Trigger-positive test results	876	517	1043
Patients with tests	113 032	60 781	83 399
Patients with abnormal test results	2801	1518	2763
Patients with trigger-positive test results	876	517	1043
Site follow-up rate, mean % (percentage-point SD),	72 (14.4)	69.2 (13.0)	67 (15.0)
Difference vs preintervention, mean (95% CI), percentage points	NA	0.36 (−5.19 to 5.90)^a^	0.89 (−6.85 to 8.63)^b^
*P* value	NA	.90	.82

Abbreviation: NA, not applicable.

^a^ Primary outcome.

^b^ Secondary outcome.

### Exploratory Outcomes

**Figure 2.**
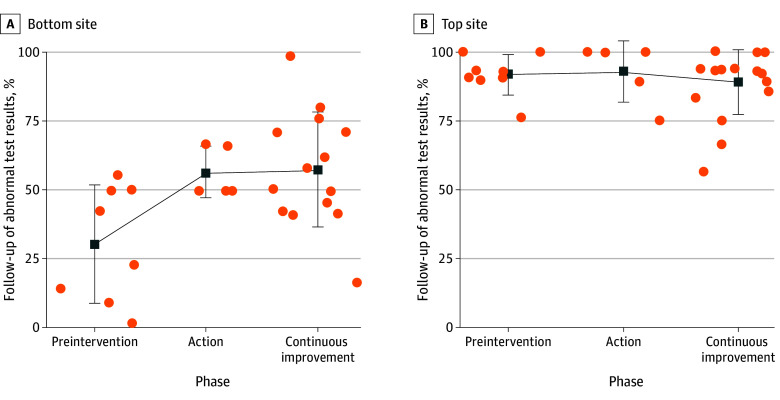
Effect of Virtual Breakthrough Series Intervention by Site Baseline Performance We conducted an exploratory analysis of change in follow-up of abnormal test results suggestive of colorectal or lung cancer by study phase, comparing the site with the lowest follow-up rate during the preintervention phase with the site with the highest rate. Percentages of follow-up for abnormal test results are shown for each month. Both sites are compared on abnormal test results suggestive of colorectal cancer. Squares indicate mean; whiskers, SD; dots, individual data points. Comparing the effect of the intervention on this electronic trigger at these sites, we found a significant effect of the study phases for the site with the lowest baseline follow-up rate (*F* = 5.49; *P* = .01) but no effect of study phases for the site with the highest follow-up rate (*F* = 0.38; *P* = .69). Specifically, the bottom site's follow-up rate increased from 27.8% in preintervention phase to 55.6% in the action phase, whereas the top site’s rate was 90.6% in the preintervention phase and 94.0% in the action phase.

The study cohort was a significant modifier of the effect of the intervention on test result follow-up (*F* = 4.61; *P* = .003). Subsequent testing and graphical inspection showed this was because of a significant effect in only cohort 2 for the lung e-trigger (eFigure 4 and eFigure 5 in Supplement 2). Comparing the effect of the intervention on this e-trigger at these sites (Figure 2), we found a significant effect of the study phases for the site with the lowest baseline follow-up rates (*F* = 5.49; *P* = .01) but no effect of study phases for the site with the highest baseline follow-up rate (*F* = 0.38; *P* = .69). Specifically, for the site with the lowest follow-up rate, the follow-up rate increased from 27.8% in the preintervention phase to 55.6% in the action phase, whereas the site with the highest follow-up rate had a rate of 90.6% in the preintervention phase and 94.0% in the action phase. This suggests a ceiling effect, in which the intervention may improve rates only at sites with low baseline performance. However, the observed changes in sites with the lowest baseline performance may also result from regression to the mean.

## Discussion

This SW-CRCT used a VBTS aimed at improving follow-up for abnormal test results suggestive of colorectal or lung cancer. Overall, we found that the VBTS intervention did not improve follow-up for abnormal test results related to diagnostic evaluation of colorectal and lung cancers. However, exploratory analysis suggest that many sites performed at relatively high levels before the intervention, with little room to improve. Thus, the intervention may offer benefits for sites with low baseline performance.

The VBTS is a useful tool for translating multifaceted interventions to clinical practice via teams working on a common goal. The exchange of interventions, barriers, and facilitators to implementation can be a valuable tool for quality improvement. In this study, multidisciplinary teams were activated to implement various interventions related to the primary drivers. This study builds on prior work using quality improvement approaches to improve clinical care.^20,21,22,23,24,25,26^ A study by Schiff et al^12^ with similar findings applied quality improvement approaches to key ambulatory malpractice risk and safety areas and found significantly improved documentation of abnormal test results, patient notification, documentation of an action or treatment plan, and evidence of a completed plan.^12^ Unlike our work, the study by Schiff et al^12^ may have been able to detect improvements more accurately due to manual EHR review. A 2014 systematic review^38^ of learning collaboratives found that most published studies reported positive findings, but it was hard to draw conclusions about the strength of these findings due to methodological limitations (eg, lack of control groups). Another review found that most learning collaboratives reported improvements in primary effect measures but cautioned about publication bias.^39^ Furthermore, of 3 CRCTs using a learning collaborative in the ambulatory setting, 2 found no significant effect of the intervention,^40,41^ and 1 found a mixture of positive and null effects.^42^ A 2024 retrospective study by Rajan et al^31^ found that implementing the VA’s Patient Aligned Care Team delivery model was associated with short-term improvement in follow-up of certain test results, but multifactorial and sustained interventions may be needed. These findings highlight the difficulty in translating multifaceted interventions into clinical practice.

Several factors may have contributed to our results. In prior work, we found that many factors impact test result follow-up, including staffing, clinician turnover, and differences in implementing best practices for certain situations (eg, tests ordered by trainees, incidental findings, tracking systems for EHR notifications, outdated contact information, absence of backup clinicians, and tests pending at discharge).^13^ Missed test results remain an intractable problem involving diverse sociotechnical factors,^43^ many of which can individually act as single-point failures, ie, when one factor’s failure can cause the entire system’s failure. The intervention may not have been able to address these single-point failures all at once.

While the virtual aspect of this intervention was an advantage during the COVID-19 pandemic, we experienced challenges when staff were reassigned to clinical care or other pandemic-related tasks. Nonetheless, teams remained committed to the study and impact of the interventions. Coaching played a crucial role in keeping teams engaged and communicating. All sites successfully implemented e-triggers they had never used before, which was a significant undertaking.^30^ However, access to data is only the first step. To improve diagnostic performance, teams must use the data in feedback loops for learning and improvement. We could not ensure that teams acted on the measures of timely follow-up of test results. To create this measurement and learning program, teams must take actionable steps on data locally and prioritize interventions by securing leadership commitment.

### Limitations

This study has limitations. First, participating teams volunteered, potentially creating selection bias of motivated teams. We cannot say with certainty that the VBTS intervention is solely responsible for any observed changes. Second, although recommendations were made about team membership, roles, and tasks, each site defined its team composition, potentially impacting outcomes. Third, this study occurred during the COVID-19 pandemic, resulting in staff reassignments and study delays for the first cohort. We also observed varying engagement across sites and time. Specifically, team size varied, and the data manager and clinical champion sometimes performed much of the effort, with limited engagement of others.

## Conclusions

In this SW-CRCT, although teams successfully implemented changes designed to reduce missed test results, the VBTS did not result in significant change in missed test results, except among low-performing sites. These findings suggest that the VBTS approach may be most helpful for organizations with low rates of test results follow-up.

## Notes

Supplement 1.
Trial Protocol and Statistical Analysis Plan

Supplement 2. **eTable 1.** Facility Complexity Descriptions **eFigure 1.** Reducing Missed Test Results Change Package **eTable 2.** Detailed Coding of Interventions Implemented by Site **eFigure 2.** Percentage of Follow-Up Abnormal Test Results for Colorectal Cancer e-Trigger Across the 3 Study Phases **eFigure 3.** Percentage of Follow-Up Abnormal Test Results for Lung Cancer e-Trigger Across the 3 Study Phases **eFigure 4.** Results of the Intervention Across Cohorts **eFigure 5.** Results of the Intervention Across Cohorts

Supplement 3.
Data Sharing Statement
