# Supplementary material for: A Virtual Breakthrough Series Collaborative for Missed Test Results: A Stepped-Wedge Cluster-Randomized Clinical Trial
Source: JAMA Netw Open. 2024 Oct 30;7(10):e2440269. doi: 10.1001/jamanetworkopen.2024.40269 (PMC11525607; doi:10.1001/jamanetworkopen.2024.40269)
Supplement: Supplement 1. — Trial Protocol and Statistical Analysis Plan [file jamanetwopen-e2440269-s001.pdf]

✓Compare
✓New Compare

Institutional Review Board for Baylor College of Medicine and Affiliated Hospitals

Protocol Number: **H-45450**
Status: Admin Mods Required
Initial Submit Date: 8/1/2019

Section Aa: Title & PI

A1. Main Title

MEASURING AND IMPROVING THE SAFETY OF TEST RESULT FOLLOW-UP - AIM 2

A2. Principal Investigator

Name:	HARDEEP SINGH	Phone:	713-794-8601
Id:	671286	Fax:	
Department:	MEDICINE: HEALTH SRVCS RESEARCH	Email:	hardeeps@bcm.edu
Center:		Mail Stn:	BCM288

A3. Administrative Contact

Name:	USMAN MIR	Phone:	
Id:	238059	Fax:	
		Email:	u238059@bcm.edu
		Mail Stn:	

A3a. Financial Conflict of Interest

Does any member of study personnel (Investigator (including investigatorâ€™s spouse and/or dependent children)) that are involved in the design, conduct, or reporting of the research have a Significant Financial Interest (SFI) that would reasonably appear to be affected by the research for which funding is sought and/or associated with an entity/business that would reasonably appear to be affected by the research?

No

Section Ab: General Information

A4. Co-Investigators

Name:	ASHLEY N.D. MEYER	Phone:	713-794-8627
Id:	161137	Fax:	713-748-7359
Department:	MEDICINE: HEALTH SRVCS RESEARCH	Email:	ameyer@bcm.edu
Center:		Mail Stn:	BCM288

Name:	TRABER GIARDINA	Phone:	713-440-4400
Id:	161560	Fax:	713-748-7359
Department:	MEDICINE: HEALTH SRVCS RESEARCH	Email:	traberd@bcm.edu
Center:		Mail Stn:	BCM288

Name:	ANDREW ZIMOLZAK	Phone:	713-440-4400
Id:	205762	Fax:	713-748-7359
Department:	MEDICINE: HEALTH SRVCS RESEARCH	Email:	zimolzak@bcm.edu
Center:		Mail Stn:	BCM288

Name:	LISA ZUBKOFF	Phone:	802-295-9363
Id:	Non-Baylor	Fax:	

Institution: White River Junction VA Medical Center Email: lisa.zubkoff@va.gov
Address: 215 N Main St. White River Junction, VT 05009

A5. Funding Source:

Organization: VA HSR&D

A6a. Institution(s) where work will be performed:

BCM: Baylor College of Medicine
Michael E. DeBakey Veterans Affairs Medical Center

A6b. Research conducted outside of the United States:

Country:
Facility/Institution:
Contact/Investigator:
Phone Number:

If documentation of assurances has not been sent to the Office of Research, please explain:

A7. Research Category:

A8. Therapeutic Intent

Does this trial have therapeutic intent?

No

A9. ClinicalTrials.gov Registration

Does this protocol/trial require registration on ClinicalTrials.gov due to it: meeting the definition of an Applicable Clinical Trial, being required under the terms and conditions of an award, or being proposed to be published in ICMJE journals?

No, this clinical is not a clinical trial, or does not meet the definition of an Applicable Clinical Trial, or does not need to be registered under the terms and conditions of an award, or is not a clinical trial with results intended to be reported in an journal belonging to the ICMJE. Registration is not required.

Section B: Exempt Request

B. Exempt From IRB Review

Not Applicable

Section C: Background Information

Improving communication is foundational to improving patient safety. Electronic health records (EHRs) can improve communication, but also introduce unique vulnerabilities. Failure to follow-up abnormal test results (missed results) is a key preventable factor in diagnosis and treatment delays in the VHA and often involves EHR-based communication breakdowns. Our work confirms that failure to follow-up abnormal results is a significant safety concern despite use of a reliable EHR. For instance, the View Alert notification system is used widely to communicate test results in the VHA's EHR, but our evaluation found that 18% of abnormal imaging and 10% of abnormal lab results were unacknowledged by providers. Furthermore, 8% and 7%, respectively, lacked timely follow-up, even when results were acknowledged.

Effective methods are needed to detect diagnostic delays and intervene appropriately. Manual techniques to detect care delays, such as spontaneous reporting and random chart reviews, have limited effectiveness, due in part to bias and lack of provider awareness of delays. They are also inefficient and cost-prohibitive when applied to large numbers of patients.

Diagnostic errors are considered harder to tackle, in part because they are difficult to measure. Rigorous measurement of diagnostic safety is essential and should be prioritized given the increasing amount of electronically available data. To create an effective measurement and learning program we must (1) ensure teams know how to take actionable steps on data and have assistance in doing so and (2) prioritize diagnostic safety at the organizational level by securing commitment from local VA leadership and clinical operations personnel. This will ensure that safety measurement will translate into action.

Multiple changes (i.e. interventions) are needed to improve follow-up of test results. There is no single intervention that works. Leveraging a multifaceted intervention, the Virtual Breakthrough Series (VBTS) model, which is based on the

Institute for Healthcare Improvement's (IHI) Breakthrough Series Collaborative model, can be used as an established method for implementing change. The IHI designed their Breakthrough Series methodology to help organizations 'close the gap' between 'what we know' and 'what we do.' A Collaborative helps bring together organizations using an 'all teach, all learn' philosophy while focusing on areas where they would like to improve. This model originally involved in-person meetings, but has been adapted to be 'virtual,' so that monthly meetings are conducted via phone. VBTS involves monthly Learning Sessions where participants are provided with education/review of a specific component of a Change Package. A Change Package is a catalogue of evidence-based practices, change concepts, and action steps/strategies that help guide improvement efforts. The VBTS sessions include learning about best practices, reviewing de-identified data, and having a facilitated discussion about successes, challenges, and implementation progress related to needed changes.

The study proposed here is part of a VA HSR&D funded grant (grant document attached in Section S). It is designed to create a novel program to develop and evaluate multifaceted socio-technical tools and strategies to help prevent, detect, mitigate, and ameliorate breakdowns in EHR-based communication that often lead to "missed" test results in the VHA. This protocol is for the second aim of this project.

Section D: Purpose and Objectives

The purpose of this study is to develop and evaluate a new program for surveillance and improvement of test results-related diagnostic safety. We will use a multifaceted measurement approach, the Virtual Breakthrough Series (VBTS) model, to account for processes of care and work systems issues as well as outcomes and implement change. We will build on our extensive experience and collaborations with several experts and stakeholders to impact surveillance of test results-related diagnostic safety within and outside the VA. '

Working with two operational partners (NCPS and VA), our specific aim is to evaluate if the "SAFER TRACKS" Intervention can reduce missed results using a stepped-wedge cluster-randomized control trial. The "SAFER TRACKS" Intervention consists of the delivery of the SAFER Change Package using a Virtual Breakthrough Series [VBTS] Collaborative supplemented with automated surveillance data on test results.

Section E: Protocol Risks/Subjects

E1. Risk Category

Category 1: Research not involving greater than minimum risk.

E2. Subjects

Gender:
Both

Age:
Adult (18-64 yrs), Geriatric (65+ yrs)

Ethnicity:
All Ethnicities

Primary Language:
English

Groups to be recruited will include:
Healthy, non-patient, normals

Which if any of the following vulnerable populations will be recruited as subjects?
Employees or lab personnel

Vulnerable populations require special protections. How will you obtain informed consent, protect subject confidentiality, and prevent undue coercion?
Some or all of the subjects are likely to be vulnerable when comparing themselves to other participating facilities due to which we will use additional safeguards to protect their rights and welfare. The facilities will be given assurance that their participation will not negatively effect them, and their individual responses will not be shared with the other subjects. We will maintain a high degree of confidentiality of their responses. Only research team members will have access to raw data. Only aggregate results, without any details that could lead to identification of a participant will be shared.

E3. Pregnant woman/fetus

Will pregnant women and/or fetuses (as described in 45 CFR 46 Subpart B) be enrolled in the research?
No

E4. Neonates

Will neonates of uncertain viability or nonviable neonates (as described in 45 CFR 46 Subpart B) be enrolled in the research?
No

E5. Children

Will children be enrolled in the research?
No

Section F: Design/Procedure

F1. Design

Select one category that most adequately describes your research:
b) Database Review

Discuss the research design including but not limited to such issues as: probability of group assignment, potential for subject to be randomized to placebo group, use of control subjects, etc.

We will use a stepped-wedge cluster-randomized control trial comparing the performance of 12 participating facilities during Pre-action (baseline) and Action (Intervention) Phases. We will measure outcomes for variable time periods in the Pre-Action and Action Phases, according to when facilities are cluster-randomized to receive the intervention. Cluster-randomization will occur at the facility level, with four facilities randomized per cluster. We will also measure outcomes in the Post-Intervention Phase for 12 months.

Aim 2 will consist of two related, continuous activities. First, we will help implement the SAFER Change Package (developed during Aim 1), using Virtual Breakthrough Series (VBTS) at participating facilities. Second, we will automatically query the corporate data warehouse (CDW) biweekly to identify possible lost to follow-up events in a pre-specified time period, using our previously developed triggers, to determine if we are able to reduce missed test results through implementation of SAFER TRACKS using VBTS.

Inclusion Criteria:

Activity 1: VBTS Teams

Activity 2: Medical records identified via CDW that have certain tests or procedures which fall under the EPRP measures (FOBT/FIT, HCV, Mammogram, AFP, DEXA Scan, Pap/HPV, Chest X-ray, and Chest CT) will be included in this study. Medical records containing certain clinical findings suspicious for breast cancer, lung cancer, bladder cancer, hepatocellular carcinoma, and colorectal cancer (CRC) will also be included in this study.

Exclusion Criteria:

Medical records that don't contain any tests, procedures, or appointments that need to be followed up on will be automatically excluded as that data will not be present in VINCI.

F2. Procedure

The SAFER TRACKS Intervention will consist of Pre-Action and Action Phases, followed by a Post-Intervention Phase, where continuous improvement and spread will occur within the facilities without support from the research team. Virtual Breakthrough Series will be facilitated and conducted by Lisa Zubkoff in order to provide a quality improvement approach that educates and encourages group learning.

During the Pre-Action Phase, the research team will conduct introductory calls to discuss an overview of what the Virtual Breakthrough Series(VBTS) sessions are, use of the change package, and what is expected from each team during monthly calls. To support this collaboration, facilities will be cluster-randomized in groups of four, so they can learn from each other during the intervention. The facilities have already formed VBTS teams (consisting of senior leadership, physicians, clinical applications coordinator, additional IT and informatics personnel, clinicians, and quality and patient safety personnel). The research team will ensure that the facilities know how to evaluate baseline test results follow-up data.

The Action Phase will include a series of group conference calls in which the research staff will present a VBTS curriculum to implement the SAFER TRACKS intervention. Much of this will build on contextual data collected in the Pre-Action Phase about process, workflow, and organizational changes. The project faculty will also provide individual coaching to teams through conference calls and e-mail. The research team will also be conducting CDW surveillance by running triggers on each site via VINCI and capturing data to determine whether the SAFER TRACKS intervention was able to reduce missed test results over the Action and Post-Intervention phase.

We will also help facilities implement trigger-based measurement of missed test results in their own data warehouse system for CDW surveillance. Implementation of up to five cancer-related triggers (lung, bladder, colorectal, hepatocellular, and breast) using the medical record data contained within the CDW will be facilitated at each of the 12 participating

sites.The analysts from the research team will send the SQL algorithm to participating sites where the site analyst will adapt and/or modify the code to run it successfully. Once the algorithm for each trigger had been finalized, manual chart reviews on a random sample of records will be conducted by the site analysts to test the trigger output. These triggers will help capture patients whose abnormal test results were not followed-up. Site analysts will run these triggers monthly and present de-identified surveillance data and discuss any trends over time during VBTS calls. Each team will share lessons learned and report on their progress. The duration of the Action phase will be 6 months for each cluster.

During the final Post-Intervention Phase (where continuous improvement and spread occur), teams will continue to implement changes as part of their usual practice for 3, 6, or 12 months depending on which cluster they belong to. We will highlight team achievements and lessons learned and shift the ownership of improvement to the facility leadership. Although no further calls or report submissions will be scheduled at this point, we will develop a sustainability tool (worksheets adapted from our previous work) to assist teams with assigning tasks, identifying responsibilities, and coordinating future facility-level activities.

Section G: Sample Size/Data Analysis

G1. Sample Size

How many subjects (or specimens, or charts) will be used in this study?

Local: 0 Worldwide: 12

Please indicate why you chose the sample size proposed:

Power analyses of primary outcomes using Hemming and Girling's method for determining sample size for a stepped-wedge cluster-randomized control trial and using ANOVAs comparing performance in Pre-Intervention versus Intervention (Action) Phases in 12 sites (assuming alpha=.05; two-tailed) show:

(a) mean improvement of percentage of patients being notified of actionable test results from 66% in the Pre- Intervention Phase (baseline) to 78% in the Intervention (Action) Phase gives 93% power to detect a significant difference in performance (additional assumptions made include: average cluster size=34 observations per quarter; number of clusters randomized at each step=4; number of steps=3; intraclass correlation=.10; 3,264 total observations); and

(b) mean reduction of patients identified as high-risk for missed follow-up of cancer related abnormal test results through electronic indicators in participating sites from 80 (per cluster per quarter) in the Pre-Intervention Phase (baseline) to 75 in the Intervention (Action) Phase, provides 94% power (additional assumptions made include: standard deviations of 30 and 25; average cluster size=70 observations per quarter; number of clusters randomized at each step=4; number of steps=3; intraclass correlation=.10; 6720 total observations). Power analysis for (b) took into account the misclassification inherent in the triggers, but utilizing prior data of all "triggered" events regardless of true positive or false positive status.

G2. Data Analysis

Provide a description of your plan for data analysis. State the types of comparisons you plan (e.g. comparison of means, comparison of proportions, regressions, analysis of variance). Which is the PRIMARY comparison/analysis? How will the analyses proposed relate to the primary purposes of your study?

The primary analyses will compare performance in the 12 participating sites in the Pre-Intervention Phase to their performance in the Intervention (Action) Phase. This will be done separately for both measures: (a) percentage of actionable test results followed up (as measured by VHA's EPRP measures) and (b) number of missed test results (determined by automated indicators through VINCI).

The Pre-Intervention Phase will include the data from the time period prior to the intervention, and the Intervention (Action) Phase will include data from the Action Phase of the intervention (excluding the 3-month, Intervention [Pre-Action] Phase).

Analyses for both measures will use univariate analyses of variance. Additionally, time series analysis will be done to assess if performance in the Post-Intervention Phase worsens over time once the intervention is carried out solely by the sites.

Section H: Potential Risks/Discomforts

H1. Potential Risks/Discomforts

Describe and assess any potential risks/discomforts; (physical, psychological, social, legal, or other) and assess the likelihood and seriousness of such risks:

During the VBTS sessions, educating and participating in group learning topics about facility operations and patient safety, there is a risk that the facility VBTS teams may experience bad feelings about a situation or their team, especially if patient harm occurred. We will mitigate these risks by being sensitive and responsive to the mood of the participants, and also emphasizing the stance of this research agenda - that it is whole systems, not individual people, that drive outcomes. The study does not place the facilities at risk of any additional harm. Small risk of confidentiality exists if the VBTS teams

disclose certain sensitive details but we will mitigate this risk by keeping data confidential (see confidentiality section below for more details). No identifying information will be used in data reporting.

For the triggers and EPRP data, we propose to collect clinical information about events that have already transpired. The study does not place human subjects at risk of any additional physical harm. There is a potential risk of psychological harm to patients if personal health information is somehow obtained by non-study personnel. All of the risks described are related to breach of confidentiality. No identifying information will be used in data reporting. We do not expect any additional significant risk (physical, social or other) or discomfort from subjects' participation in these activities over and above what is already present in the normal course of events unrelated to research. Because of the guidelines in place ensure confidentiality, these harms are very unlikely.

H2. Data and safety monitoring plan

Do the study activities impart greater than minimal risk to subjects?

No

H3. Coordination of information among sites for multi-site research

Is the BCM Principal Investigator acting as the SPONSOR-INVESTIGATOR for this multi-site research?

No or Not Applicable

Is BCM the COORDINATING CENTER for this multi-site research?

No or Not Applicable

Section I: Potential Benefits

Describe potential benefit(s) to be gained by the individual subject as a result of participating in the planned work.

Facilities could experience improvement in test results follow-up and reduced delays in care.

Describe potential benefit(s) to society of the planned work.

Improving test result communication is foundational to improving patient safety. This multifaceted intervention, based on the eight dimension sociotechnical model, is a unique approach in the area of test results follow-up. In addition, we are studying novel concepts such as whether a VBTS model can be effectively used for reducing missed test results in VA settings. If our aims are achieved, our study could put into place novel surveillance and improvement strategies for test results follow-up and identify care delays more efficiently.

Do anticipated benefits outweigh potential risks? Discuss the risk-to-benefit ratio.

There are overwhelming benefits from identifying care delays in terms of preventing excess morbidity and mortality and reducing associated costs. These potential benefits outweigh the very minor risks to participants.

Since anticipated risk or discomfort expected from participation is low, the benefit of information gained from the project should substantially outweigh the risks associated with study participation. There are also societal benefits of improving the quality of care and improving the processes of improving test-result related communication.

Section J: Consent Procedures

J1. Waiver of Consent

Will any portion of this research require a waiver of consent and authorization?

Yes

Please describe the portion of the research for which a waiver is required. (Example: chart review to determine subject eligibility)

We will be running triggers to get counts of missed/delayed diagnosis across VA facilities using VINCI to retrieve data by running our cancer-related trigger algorithms. We will require a consent waiver as well as a HIPAA authorization for the entire study.

Explain why the research and the use or disclosure of protected health information involves no more than minimal risk (including privacy risks) to the individuals.

The use or disclosure of protected health information involves no more than minimal risk to the individuals in this study as it will only be used to extrapolate trigger output data. Thus, protected health information will only be used to assess the effectiveness of health information technology to identify patients with cancer related diagnostic treatment delays.

Explain why the waiver will not adversely affect the privacy rights and the welfare of the research subjects.

The waiver will not adversely affect the privacy rights or welfare of the individuals. This research study is limited to accessing, collecting, and analyzing existing medical record information. There are no physical or psychological risks to the human subjects (i.e. the respective patients) associated with the conduct of this research study. We will only be

collecting data from the patient record in order to assess facility performance. This data will include 1) cancer-related symptoms without follow-up; 2) new abnormal test results without follow-up; 3) time to follow-up; 4) patient demographics (date of birth, comorbid conditions, date of visits or missed appointments, and consultation, imaging, laboratory, and pathology result data).

Explain why the research could not practicably be conducted without the waiver and could not practicably be conducted without access to and use of the protected health information.

It would not be feasible to obtain consent for medical records that are contained in the CDW to assess facility performance. Pulling out medical record data is the only way we can perform this kind of research. It is not possible to conduct this research study unless we are able to identify and access selected medical records from the 12 sites.

In summary, this research activity (i.e., retrieving medical record data) could not practicably be conducted without a waiver of the HIPAA authorization requirement. Consistent with the 'minimum necessary standard' of the HIPAA privacy rule, we will only access and collect the specific health information necessary to complete this research study.

Describe how the research could not practicably be carried out without using the collected identifiable biospecimens in an identifiable format.

N/A

Describe how an adequate plan exists in order to protect identifiers from improper use and disclosure.

Medical record data will be assigned a unique code. Computer entry, data coding, and analyses will use only the unique codes. Both the anonymized health information and the information linking the research code numbers to the patients' identifies will be stored in a secure manner (e.g., locked file cabinet behind locked doors, password protected database behind the VA firewall) accessible only to the research study investigators. We hereby provide our assurance that the protected health information will not be reused or disclosed to any other person or entity, except as required by law.

Describe how an adequate plan exists in order to destroy identifiers at the earliest opportunity consistent with conduct of the research, unless there is a health or research justification for retaining the identifiers or such retention is otherwise required by law.

Research Investigator files will be destroyed six years after the end of the fiscal year when the research project has been completed per Records Schedule DAA-0015-2015-004, Section 7.6, Research Investigator Files.

Describe how adequate written assurances exist in order to ensure that the PHI will not be reused or disclosed to (shared with) any other person or entity, except as required by law, for authorized oversight of the research study, or for other research for which the use or disclosure of the PHI would be permitted under the Privacy Rule.

PHI will not be reused or disclosed to any other person or entity except as required by law or research oversight. It is understood by the PI that data will not be used or shared with others outside the scope of this research study, and removal of access to research study data will be accomplished for all study personnel when they are no longer part of the research team. All research team members are trained in human subjects protection, and all data stays either on the password and firewall-protected M drive at the IQuEST Center or in a locked storage cabinet behind two locked doors. Research team members will follow the written protocol for protection of PHI that exists as part of this protocol and the human subjects protection training they complete.

Information from health records such as diagnoses, progress notes, medications, lab or radiology findings, etc.

Yes

Specific information concerning alcohol abuse:

No

Specific information concerning drug abuse:

No

Specific information concerning sickle cell anemia:

No

Specific information concerning HIV:

No

Specific information concerning psychiatry notes:

No

Demographic information (name, D.O.B., age, gender, race, etc.):

Yes

Full Social Security #:

Yes

Partial Social Security # (Last four digits):

Yes

Billing or financial records:
No

Photographs, videotapes, and/or audiotapes of you:
No

Other:
No

Will additional pertinent information be provided to subjects after participation?
No

If No, explain why providing subjects additional pertinent information after participation is not appropriate.
Given that there is no clinical intervention in this project (database review only) it would not be appropriate to provide additional information. The data collected from this study will be analyzed, summarized and presented as summary statistics (i.e. with no identifying features) in scientific abstracts and manuscripts.

J1a. Waiver of requirement for written documentation of Consent

Will this research require a waiver of the requirement for written documentation of informed consent?
No

J2. Consent Procedures

Who will recruit subjects for this study?
PI
PI's staff

Describe how research population will be identified, recruitment procedures, any waiting period between informing the prospective participant and obtaining consent, steps taken to minimize the possibility of coercion or undue influence and consent procedures in detail.
Dr. Singh has successfully recruited 12 VA facilities through the many national leadership roles he's held and currently holds (see site confirmation emails attached in Section S). The site champions and VBTS session participants have already been identified during Aim 1 of the study. The personnel at each site who participated in the stakeholder interviews to develop the change package in Aim 1, will make up the facility VBTS teams. No additional recruitment activity will occur.

Are foreign language consent forms required for this protocol?
No

J3. Privacy and Intrusiveness

Will the research involve observation or intrusion in situations where the subjects would normally have an expectation of privacy?
No

J4. Children

Will children be enrolled in the research?
No

J5. Neonates

Will non-viable neonates or neonates of uncertain viability be involved in research?
No

J6. Consent Capacity - Adults who lack capacity

Will Adult subjects who lack the capacity to give informed consent be enrolled in the research?
No

J7. Prisoners

Will Prisoners be enrolled in the research?
No

Section K: Research Related Health Information and Confidentiality

Will research data include identifiable subject information?

Yes

Information from health records such as diagnoses, progress notes, medications, lab or radiology findings, etc.
No

Specific information concerning alcohol abuse:
No

Specific information concerning drug abuse:
No

Specific information concerning sickle cell anemia:
No

Specific information concerning HIV:
No

Specific information concerning psychiatry notes:
No

Demographic information (name, D.O.B., age, gender, race, etc.):
Yes

Full Social Security #:
Yes

Partial Social Security # (Last four digits):
Yes

Billing or financial records:
No

Photographs, videotapes, and/or audiotapes of you:
No

Identifiable biospecimens
No

Other:
No

At what institution will the physical research data be kept?
All data in paper form (i.e., regulatory binder and data collection forms) associated with this protocol will be stored in a locked storage cabinet in a locked room, room 121 (data storage room), at the Houston IQuESt Center of Innovation at the MEDVAMC, Nabisco Building, McGovern Campus (2450 Holcombe Blvd., Suite 01Y, Houston, TX 77021)

How will such physical research data be secured?
Physical data will be stored in a locked storage cabinet in a locked room (221) at the IQuESt Center in Houston, TX. Only research team members with the correct passcode and keys will be able to access the room and the cabinet.

At what institution will the electronic research data be kept?
All electronic data will be stored on a drive dedicated to research on a server at the Houston IQuEST Center (2450 Holcombe Blvd., Suite 01Y, Houston, TX 77021).

Such electronic research data will be secured via BCM IT Services- provided secured network storage of electronic research data (Non-Portable devices only):
No

Such electronic research data will be secured via Other:
Yes, (describe below):
Data will be stored on a VA server at the Houston IQuESt Center of Innovation. This server is controlled by the VA facility and resides within the VA domain and firewall set up and can only be accessed through a password protected, encrypted computer behind VA firewall. This server is physically located in a server room in a VA-controlled facility, locked by card access and monitored by camera. Data will remain within the project-specific subfolder (\\r02.med.va.gov\Research\HOU\Production\Data\Housrd\Projects\PSCI\IIR (InSTRuCt). Access to the data will be granted by the data administrator via VHA-domain access. Data will not leave the VA. No portable media will be used for this protocol. No thumbdrives, flashdrives, portable hard devices, or laptop computers will be used for this protocol.

All participants will be assigned a unique identifier number. Individual information used to facilitate scheduling of study

activities (e.g., potential participants' telephone numbers and/or email addresses) will not be linked to these identifier numbers and will be kept in a separate location. No PHI will be collected for the VBTS intervention.

Will there be anyone besides the PI, the study staff, the IRB and the sponsor, who will have access to identifiable research data?

Yes, identify the classes of the persons:
People who ensure quality from the institution where the research is being done, federal and other regulatory agencies will have access to all of the research data.

Please describe the methods of transmission of any research data (including PHI, sensitive, and non-sensitive data) to sponsors and/or collaborators.

PHI will not be transmitted to sponsors or collaborators.

Will you obtain a Certificate of Confidentiality for this study?

No

Please further discuss any potential confidentiality issues related to this study.

Protection of patient data: To retrieve medical records, the following HIPAA identifiers will be utilized - SSN. Data extracted will be assigned a unique identifier code specific to this study, which will not be linked to any other identifier (for instance, the study identifier will not simply be a scrambled version of another existing identifier such as a medical record number). Computer entry, data coding, and analyses will use only these unique codes to identify records. No identifying information will be used in data reporting.

There is no plan to disclose or otherwise grant access to VINCI/CDW data to entities outside or within VHA. Only authorized personnel will have access to the data and personnel who no longer need the information will have their access removed.

Research records, including identifiers will be destroyed 6 years after cutoff (at the end of the fiscal year) after completion of the research project, but may be retained longer if required by other federal regulations or sponsor archive requirement.

It is understood by the PI that data will not be used or shared with others outside the scope of the research study.

Removal of access to research study data will be accomplished for all study personnel when they are no longer part of the research team.

An Accounting of Disclosure (AOD) will be created and maintained for any disclosure of individually identifiable information (III) outside the VA. The manual spreadsheet will include the date of the disclosure, nature or description of the III disclosed, purpose of each disclosure and the name and address of person or agency to which the disclosure was made.

Section L: Cost/Payment

Delineate clinical procedures from research procedures. Will subject's insurance (or subject) be responsible for research related costs? If so state for which items subject's insurance (or subject) will be responsible (surgery, device, drugs, etc). If appropriate, discuss the availability of financial counseling.

If subjects will be paid (money, gift certificates, coupons, etc.) to participate in this research project, please note the total dollar amount (or dollar value amount) and distribution plan (one payment, pro-rated payment, paid upon completion, etc) of the payment.

Dollar Amount:

Distribution Plan:

Section M: Genetics

How would you classify your genetic study?

Discuss the potential for psychological, social, and/or physical harm subsequent to participation in this research. Please discuss, considering the following areas: risks to privacy, confidentiality, insurability, employability, immigration status, paternity status, educational opportunities, or social stigma.

Will subjects be offered any type of genetic education or counseling, and if so, who will provide the education or counseling and under what conditions will it be provided? If there is the possibility that a family's pedigree will be presented or published, please describe how you will protect family member's confidentiality?

Section N: Sample Collection

None

Section O: Drug Studies

Does the research involve the use of ANY drug* or biologic? (*A drug is defined as any substance that is used to elicit a pharmacologic or physiologic response whether it is for treatment or diagnostic purposes)

No

Does the research involve the use of ANY gene transfer agent for human gene transfer research?

No

O1. Current Drugs

Is this study placebo-controlled?

No

Will the research involve a radioactive drug?

No

Section P: Device Studies

Does this research study involve the use of ANY device?

No

Section Q. Consent Form(s)

None

Section R: Advertisements

None

Statistical Analysis Plan

Power analyses of primary outcomes will be done using Hemming and Girling's method[35,36] for determining power for SW-CRTs comparing performance in pre-intervention versus action phases (assuming α=0.05; two-tailed; intraclass correlation=0.10). Assuming 12 sites for feasibility, a mean improvement of follow-up of abnormal test results from 56% in pre-intervention to 67% in action phase gives 82% power to detect a significant difference in colorectal cancer evaluation-related e-trigger performance.[6] Similarly, a mean improvement of follow-up of abnormal test results from 61% in pre-intervention to 73% in the action phase gives 89% power to detect a significant difference in lung cancer evaluation-related e-trigger performance.[33]

We will compare e-trigger rates among the three phases using linear mixed-effects models[37] to compute estimated mean differences in follow-up with 95% confidence intervals. Models will compare pre-intervention vs. action phase (primary outcome) and pre-intervention vs. continuous improvement (secondary outcome) by including study phase as a fixed effect, study month (number 1–26) as a fixed effect to account for calendar time, and individual site as a random effect.

For the exploratory analysis, we will use ANOVA to model the interaction of cohort and study phase and their effect on the percentage of test result follow-up. All tests will be two-tailed, and a p-value <0.05 will be considered significant. Analyses will be performed using STATA version 11 (College Station, Texas) and R version 4.2.1 (R Project for Statistical Computing) with the "lme4" package.[38]
