# Supplementary material for: A Virtual Breakthrough Series Collaborative for Missed Test Results: A Stepped-Wedge Cluster-Randomized Clinical Trial
Source: JAMA Netw Open. 2024 Oct 30;7(10):e2440269. doi: 10.1001/jamanetworkopen.2024.40269 (PMC11525607; doi:10.1001/jamanetworkopen.2024.40269)
Supplement: Supplement 2. — eTable 1. Facility Complexity Descriptions eFigure 1. Reducing Missed Test Results Change Package eTable 2. Detailed Coding of Interventions Implemented by Site eFigure 2. Percentage of Follow-Up Abnormal Test Results for Colorectal Cancer e-Trigger Across the 3 Study Phases eFigure 3. Percentage of Follow-Up Abnormal Test Results for Lung Cancer e-Trigger Across the 3 Study Phases eFigure 4. Results of the Intervention Across Cohorts eFigure 5. Results of the Intervention Across Cohorts [file jamanetwopen-e2440269-s002.pdf]

Supplemental Online Content

Zubkoff L, Zimolzak AJ, Meyer AND, et al. A Virtual Breakthrough Series collaborative for missed test results: a stepped-wedge cluster-randomized clinical trial. *JAMA Netw Open*. 2024;7(10):e2440269. doi:10.1001/jamanetworkopen.2024.40269

eTable 1. Facility Complexity Descriptions
eFigure 1. Reducing Missed Test Results Change Package
eTable 2. Detailed Coding of Interventions Implemented by Site
eFigure 2. Percentage of Follow-Up Abnormal Test Results for Colorectal Cancer e-Trigger Across the 3 Study Phases
eFigure 3. Percentage of Follow-Up Abnormal Test Results for Lung Cancer e-Trigger Across the 3 Study Phases
eFigure 4. Results of the Intervention Across Cohorts
eFigure 5. Results of the Intervention Across Cohorts

This supplemental material has been provided by the authors to give readers additional information about their work.

© 2024 Zubkoff L et al. *JAMA Network Open*.

eTable 1. Facility Complexity Descriptions*

Complexity Level	Description
Highest complexity (1a)	Facilities with:<br>• high volume<br>• high risk patients<br>• most complex clinical programs<br>• large research and teaching programs
High complexity (1b)	Facilities with:<br>• medium-high volume<br>• high risk patients<br>• many complex clinical programs<br>• medium-large research and teaching programs
Mid-high complexity (1c)	Facilities with:<br>• medium-high volume<br>• medium risk patients<br>• some complex clinical programs<br>• medium sized research and teaching programs
Medium complexity (2)	Facilities with:<br>• medium volume<br>• low risk patients<br>• few complex clinical programs<br>• small or no research and teaching programs
Low complexity (3)	Facilities with:<br>• low volume<br>• low risk patients<br>• few or no complex clinical programs<br>• small or no research and teaching programs

*Adapted from National Academies of Sciences, Engineering, and Medicine 2020. *Facilities Staffing Requirements for the Veterans Health Administration Resource Planning and Methodology for the Future*. Washington, DC: The National Academies Press. https://doi.org/10.17226/25454.

© 2024 Zubkoff L et al. *JAMA Network Open*.

eFigure 1. Reducing Missed Test Results Change Package

Reducing Missed Test Results Change Package

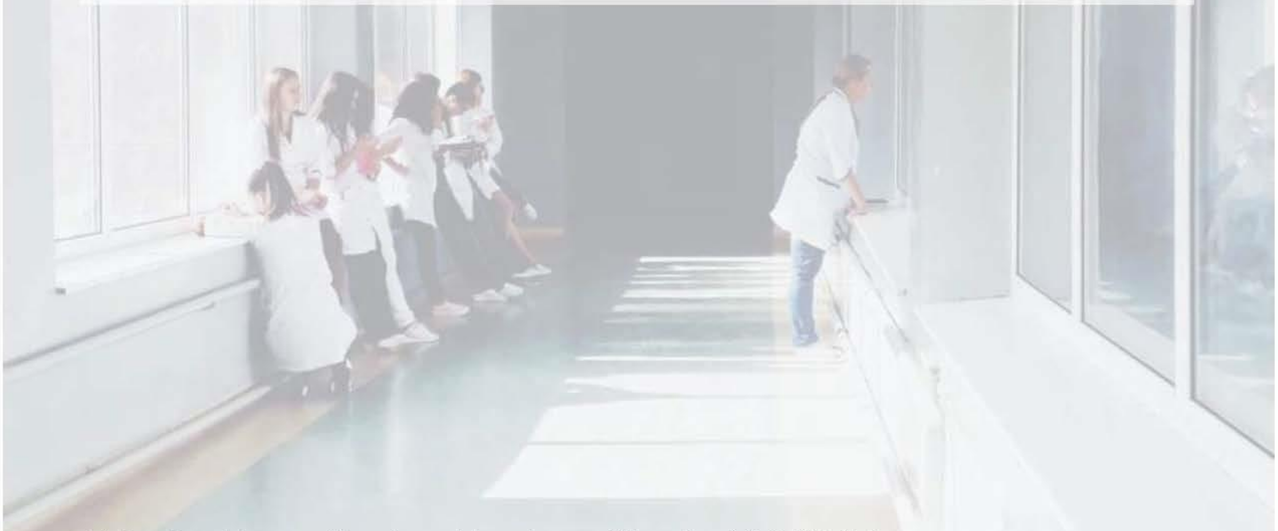

Principal Investigator: Hardeep Singh, MD MPH
InSTRuCt Team: Daniel Murphy, MD MBA, Traber Giardina, PhD, Andrew Zimolzak, MD, Lisa Zubkoff, PhD, Umair Mushtaq, MS, Umber Shahid, PhD, Sahar Memon, MPH MS

VA HEALTH CARE | Defining EXCELLENCE in the 21st Century

HSR&D
VA Health Services Research & Development Service

© 2024 Zubkoff L et al. *JAMA Network Open*.

TABLE OF CONTENTS

Introduction 3-4
Figure 1: The Model for Improvement… 3
Figure 2: Post-Analytic Process Map… 5
Figure 3: InSTRuCT Driver Diagram 6
Change Package
1. Enhancing Patient Engagement with Test Results
   a. Increase Access to Test Results 7
   b. Educate patients on expectations of the test result and follow-up process.8
   c. Increase Patient Comprehension of Test Results 9
2. Improving Situational Awareness Among All Providers and Care Teams
   a. Prevent Provider EHR Notification Fatigue 10
   b. Ensure Effective Teamwork in Management of Test Results 11
   c. Create a Support Structure to Facilitate Test Results Review and Follow-Up 12
   d. Facilitate Time for Providers to Review and Act Upon Test Results 12
3. Implement Processes to Close the Loop on Test Results Reporting and Follow-Up
   a. Ensure Providers and Staff Have Necessary Patient Contact Information for Fail-Safe Communication 13
   b. Monitor for Breakdowns in Test Results Review and Communication...... 14
   c. Employ Standardized and Fail-Safe Processes and Policies for Communicating Abnormal Test Results to Providers 15
   d. Use EHR Features to Support Closing the Loop on Test Results 16
Change Package Development 17
Definitions 18-19
References 20-21
Acknowledgements… 22

Additional Documents
Alerts Checklist… 10
CTR Toolkit… 11, 15
InSTRuCt e-Trigger Manual… 14

© 2024 Zubkoff L et al. *JAMA Network Open*.

INTRODUCTION:

Improving communication is foundational to improving patient safety. Failure to follow-up abnormal test results ("missed results") is a key preventable factor in diagnosis and treatment delays and remains a significant safety concern despite the use of a comprehensive and reliable Electronic health records (EHR). A foundational concept for ensuring patient safety is closed-loop communication, which involves acknowledging receipt of information and clarifying accuracy of that information with the message sender.

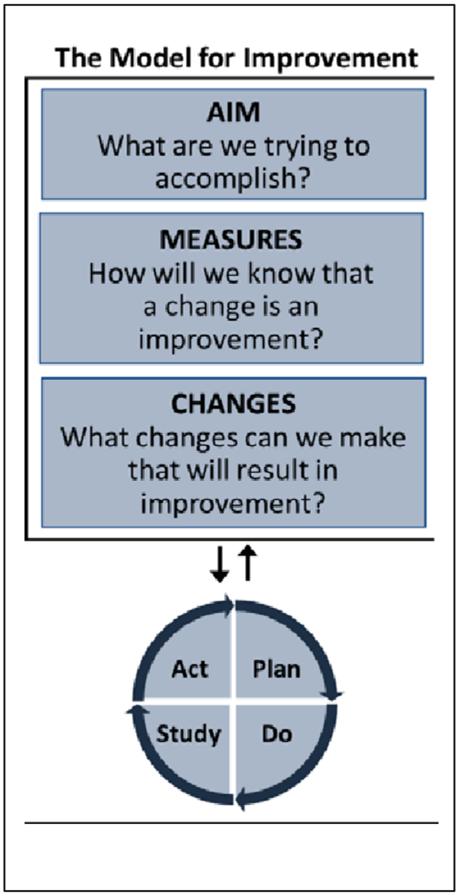

This change package aims to help health care organizations implement practices and processes to reduce missed test results. It includes patient-facing, provider-facing, and system-facing practices developed from various toolkits and guidance documents. Subject matter expert contributions and evidence-based practices have been used to develop the content of this change package. It is intended to guide transformation efforts and can be complementary to other evidence-based tools and resources. It is meant to be used as a tool to make patient care safer and improve the follow-up of test results, and can be used by any facility, in or out of the original collaborative. While this change package recommends many strategies, organizations should determine which ones they need to prioritize and focus on. **Recommendations will apply to all health care organizations and will apply regardless of the type of electronic health record being used.**

How to Use this Change Package:

This change package includes a menu of strategies, specific actionable steps, and change approaches that any VA health care facility can implement based on identified needs for improving safety of test results communication. The Model for Improvement (Figure 1) can be used to select and test changes to see if they result in improvement. A driver diagram is a visual depiction of a theory behind an improvement effort. The InSTRuCt Driver Diagram (Figure 3) displays the relationship of this global aim to the primary drivers that contribute to achieving that aim and the subsequent action steps that are necessary to achieve the primary drivers. It clearly highlights the progression of steps an organization should take towards reduction of missed test results The users are encouraged to select the action steps and build new processes that allow them to fulfill the primary drivers. Much of the success or failure will depend on assigning appropriate roles and responsibilities within teams implementing the change package. Before using this change package, users baseline data (EPRP and Triggers) should be evaluated to determine the change approach that would be the most meaningful and appropriate. Trigger algorithms will be sent over by the Houston research team and all VA sites should already have access to EPRP data and can run reports using the Combined Measure Master Report. Our recommendation would be to pick a minimum of **one** change approach from each of the 3 categories.

© 2024 Zubkoff L et al. *JAMA Network Open*.

Process map of test result follow-up activities

Timely and reliable testing is critical to diagnostic safety. Breakdowns in the testing process (Figure 2: Post Analytic Process Map) can lead to diagnostic errors, which delay appropriate treatment, lead to poor patient outcomes, decrease patient satisfaction, and increase malpractice litigation.

The testing process can be divided into three key phases:

1. The **pre-analytic phase** involves activities related to placing test orders, communicating those orders to the diagnostic center or draw station, collecting and preparing samples, and transporting and storing those samples.

2. The **analytic phase** involves activities between when samples start processing and when results become finalized.

3. The **post-analytic phase** involves communication of results to clinicians, who then discuss results with patients and take appropriate action.

Acting on abnormal test results and communicating results to patients occurs in the post-analytic phase and are prone to breakdowns in care. For example, lack of accurate patient contact information within a patient's record may cause difficulty in reaching a patient to discuss and arrange follow up after abnormal test results return. Similarly, if EHRs do not properly identify and route results to the provider responsible for follow-up or facilitate coverage of providers who are unavailable (e.g., resident-ordered tests or tests ordered by physicians who are out of the office), results might not reach the appropriate individual to arrange follow-up action in a timely manner. Finally, processing messages in interruptive environments and information overload from excessive inbox messages may impact providers' situational awareness, causing important information within these messages to be inadvertently missed. An understanding of the provider's actions that occur in this phase are essential to implementing interventions to reduce breakdowns and improve patient safety. Figure 2 and this project are focused on the post-analytic phase.

© 2024 Zubkoff L et al. *JAMA Network Open*.

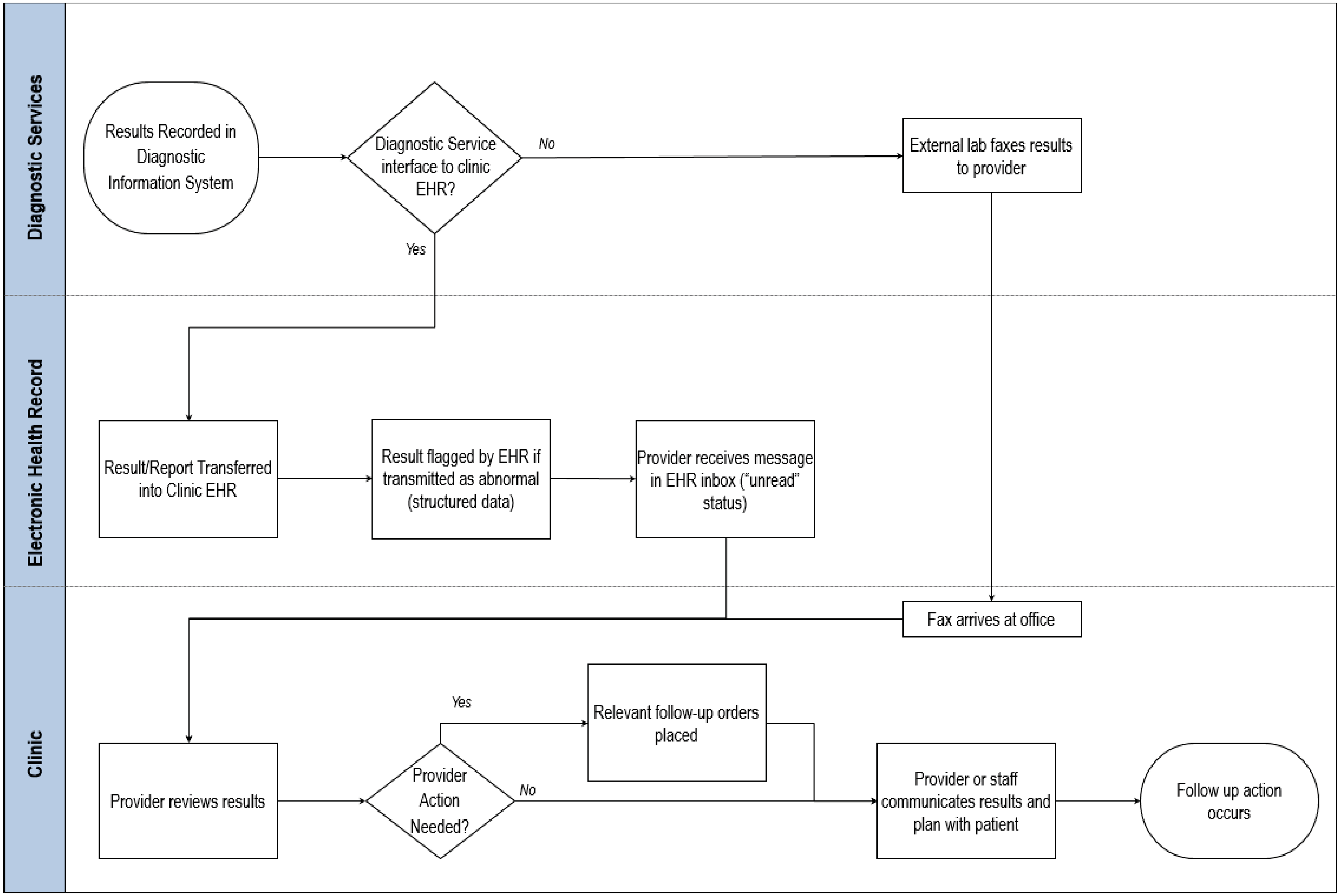

Figure 2: Post-Analytic Process Map

© 2024 Zubkoff L et al. *JAMA Network Open*.

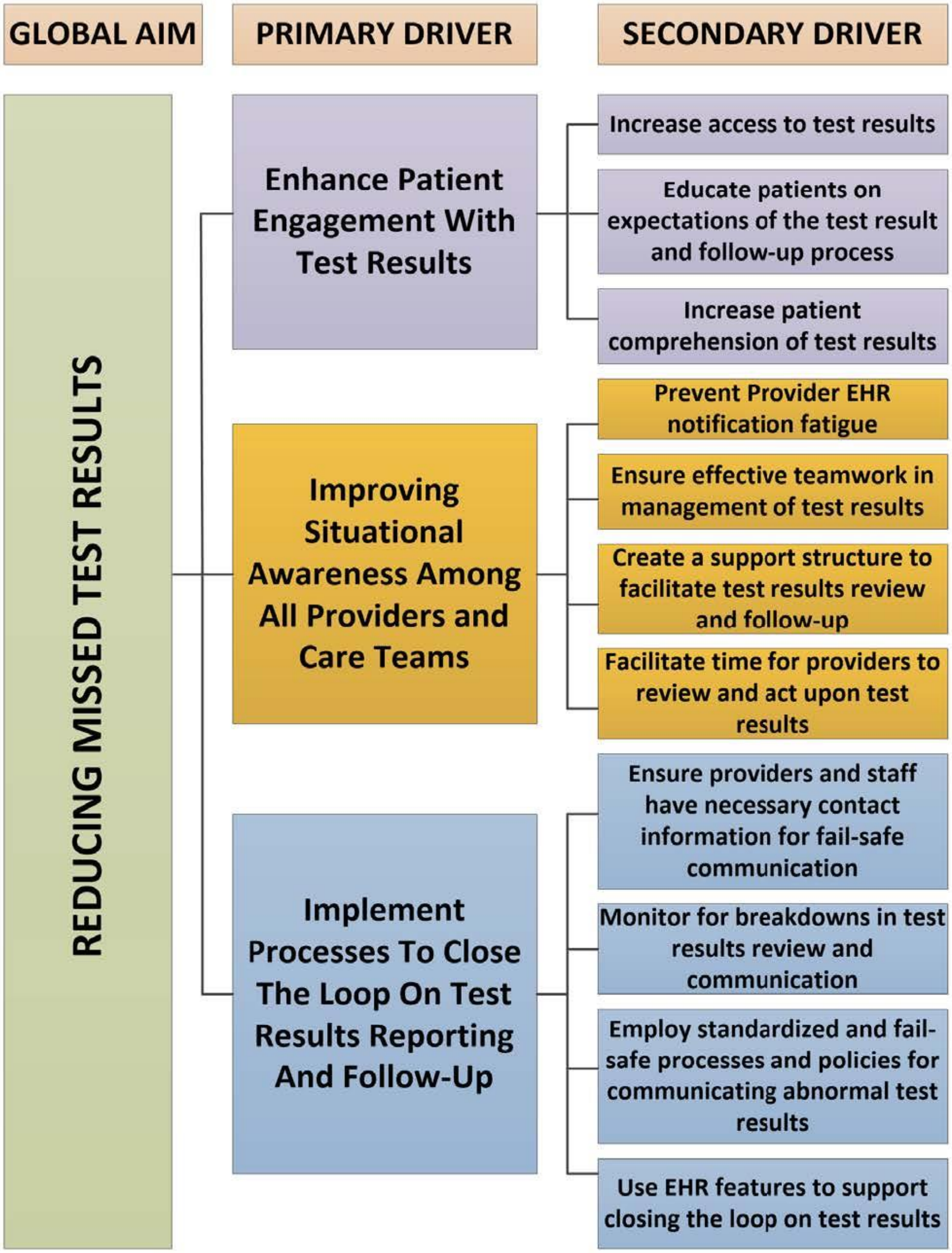

Figure 3: InSTRuCt Driver Diagram

© 2024 Zubkoff L et al. *JAMA Network Open*.

1. Enhancing Patient Engagement with Test Results

A. Increase Access to Test Results

The introduction of electronic patient portals offers many benefits to patients, including faster and more direct access to health information, such as test results, and the ability to easily communicate with health care providers. Patient access to their health information may improve self-management, create opportunities for improved partnership, and enhance patients' satisfaction and quality of care.[1-6]

The VA currently has procedures to release test results directly to patients with a time delay for imaging and path.

The patient portal (by providing access to clinical notes and test results) can increase patient engagement[5]. There is growing evidence that engaged patients are more likely to be involved in patient safety[7]. Access to test results is one of the most popular features of the patient portal[8, 9]. About 20% of patients are viewing their test results within 8 hours of release from the EHR to the patient portal[10]. Timely access to test results and encouragement[11, 12] to use the portal to access health information may provide opportunities for patients to identify delayed or missed test result follow-up.[13]

Of note, patients' preferences for test result access might change depending on type and purpose of test (routine monitoring vs. acute diagnostic). Patient characteristics and medical history should also be considered.[3, 14-16]

Step 1: Implement efforts to encourage patients to use patient portal.

Change Approach:

- At check-in for every visit, staff confirms whether patients are currently signed up for and using the portal. If not, patients should receive information to sign up (forms, point of contact, value of the portal for accessing test results and personal health information) and be encouraged to use it. Consider a designated contact to help patients sign up.
- Institutional campaigns advertising the available beneficial features of portals. (e.g. posters in rooms)
- A designated point of contact for patients to reach out to for questions and concerns regarding My HealtheVet (MHV). (MHV contact or patient services)

Step 2: Identify and record patients' preferred mechanisms for communication

Change Approach:

Patient should be asked their preference for communication of test results at every visit (along with any verification already taking place at check in). "*How would you like to be notified of your test results*? *Here are the options:* …" or "*Is* My HealtheVet *still your preferred communication option?"*

Step 3: Ensure use of training materials for portal use

Change Approach:

Patients should be trained on portal use and made aware of how to access in-person (MHV contact or patient services) or online training (here). Training currently available online for following items:

- How to access the portal.
- Features available in the three different MHV account types.
- How to upgrade account to one with additional features.
- Visual instructions showing how to access test results within MHV.
- How to reset passwords.

© 2024 Zubkoff L et al. *JAMA Network Open*.

1. Enhancing Patient Engagement with Test Results

B. Educate Patients on Expectations of the Test Result and Follow-Up Process

Patients should be aware of follow-up processes.

Based on current policy to send out test results to patients via My HealtheVet, clinicians should anticipate that patients will receive their results electronically and prepare patients accordingly. This should include explaining the reason the test was ordered, when to expect the result, and who and how to contact for questions.[14]

Lack of information related to testing and test result management along with limited collaboration between patients and clinicians have been perceived as having negative impacts on patient satisfaction and involvement in their care.[1]

ACTION STEPS

Step 1: Improve communication with patients about why tests have been ordered and make patients aware of who to contact when there are challenges in following up on abnormal results[17]

Change Approach:

When explaining to patients why tests are being ordered, provide contact information (phone and/or secure message of a clinician, nurse, staff member) for any additional questions about testing and test results that may arise.

Step 2: Increase awareness of when to expect results[17]

Change Approach:

Clinicians should clarify expectations regarding test result notification – this can be done in-person at the time of the visit, via email, and/or promotional fliers.

Step 3: Increase awareness of "no news is not necessarily good news"[17, 18]

Change Approach:

In conjunction with Step 2, develop educational procedures and institutional campaigns to encourage patients to follow-up on test results when they have not heard back within a certain time frame – No News is Not Good News.

© 2024 Zubkoff L et al. *JAMA Network Open*.

1. Enhancing Patient Engagement with Test Results

C. Increase Patient Comprehension of Test Results

Patients' access to test results does not guarantee that patients can use or understand that information. Some patients may have lower health literacy, numeracy, or graphical literacy which can impact their ability to understand[19] and use their health information.[14, 19, 20] EHRs may present test result data in a manner that make it difficult for patients to understand, including absence of additional or contextual information.[19] Some patients will experience uncertainty when accessing their test results and will turn to the internet to better understand their results; others may contact their doctor for additional information.

ACTION STEPS

Step 1: Make test results easier to understand

Change Approach:

Clinicians should anticipate that patients may not be able to understand their results as currently displayed in MHV.

- Acknowledge that it may be hard to understand test results in MHV and explore strategies to address patient understanding of results transmitted in the portal, including the following strategies:
    - Provide access to high-quality, vetted educational websites (ideally linked in the portal).[14] Websites should explain the test and its meaning at an appropriate reading level.
    - Provide access to educational services, including training patients on how to access their results in-person or via the online training (Section A-3), vetted resources to help understand the test result[14], and access to a person who can explain the test and the results when necessary (the follow-up contact person in Section 1-B, nurse, or clinician).
    - Provide a written interpretation along with test results.

© 2024 Zubkoff L et al. *JAMA Network Open*.

2. Improving Situational Awareness Among All Providers and Care Teams

A. Prevent Provider EHR Notification Fatigue[21-23]

Electronic health records (EHR), and in particular EHR inboxes such as the *Notifications* window, have helped to facilitate the exchange of clinical information. While this helps to disseminate information about patient care between clinical team members, transmission of duplicate or irrelevant messages can overwhelm and distract providers. Excessive numbers of notifications can paradoxically lead to decreased situational awareness as providers become inundated with information and become less able to focus on important and actionable information.

Providers should acquire a good understanding of simple and well-designed tools used to manage test results. Since most test results are delivered via EHR inboxes, it is essential for them to understand EHR inbox features and test result processing workflows.

This section provides suggestions to limit overloading providers with excessive numbers of EHR inbox notifications that have minimal impact on patient care, as well as ensure physicians are properly trained to process EHR-delivered test results.

ACTION STEPS

Step 1: Each clinician should optimize their own EHR notification settings to minimize information overload[23]

Change Approach:

EHR notification settings should be configured to avoid automatically sending duplicate notifications (e.g., abnormal lab panel and abnormal lab value as separate notifications)

Notifications deemed to require no action and have no clinical relevance to patient care should be disabled.

Establish a committee or task force that includes clinicians to evaluate the importance of certain EHR notifications, and disable those that are determined to be not relevant to care.

Step 2: Teach providers how to efficiently manage EHR notifications[23]

Change Approach:

Clinician EHR training should include instruction on managing test results in the EHR inbox, including features present in the EHR inbox to facilitate test result management and local testing process workflows

Step 3: Implement strategies to limit EHR inbox notifications to those that are clinically relevant (All VA clinicians and facilities should see this (View Alerts checklist)

Change Approach:

Measure daily notification burden to identify clinicians who may be overwhelmed with them, and intervene to provide them relief. You may need to speak to your local IT support for that.

Educate clinicians on proper messaging etiquette, including avoidance of generating and forwarding of FYI-only messages that are not expected to impact patient care.

© 2024 Zubkoff L et al. *JAMA Network Open*.

2. Improving Situational Awareness Among All Providers and Care Teams

B. Ensure Effective Teamwork in Management of Test Results

Coordination is an important component of clinical care; but to be effective, team members must have clearly delegated tasks, roles, and responsibilities. Poorly defined roles and responsibilities can lead to delays in care. For instance, a subspecialist receiving test results may expect the patient's primary care provider to follow up, while the primary care provider may expect the subspecialist, who ordered the test, to follow up. This diffusion of responsibility may lead to inaction by both providers and lead to delays in patient care.[24]

This section will help you structure teams and policies to assist in the management of test result review, processing, and communication.

ACTION STEPS

Step 1: Clarify delegation of tasks, roles, and key responsibilities related to test results.[18]

Change Approach:

Ensure that responsibility of test result follow up is clearly defined and implemented (e.g., policy that ordering provider is responsible for test result communication to patients and arranging follow up, unless and until responsibility is transferred and accepted by another provider). To address this, facilities should follow responsibility procedures outlined in the VHA Directive 1088.

Ensure that resident-ordered and part-time clinician-ordered tests have a mechanism to ensure follow up action even when the resident or part-time clinician is not in clinic. See Escalation procedures implemented at one facility.

Step 2: Establish protocols for delegating management of notifications to other care team providers in order to ensure everyone is working at the top of their license.

Change approach:

Staff protocols can help off load lower priority messages so physicians can focus on higher priority messages such as test results.[25-27]

© 2024 Zubkoff L et al. *JAMA Network Open*.

2. Improving Situational Awareness Among All Providers and Care Teams

C. Create a Support Structure to Facilitate Test Results Review and Follow-Up[18, 28]

Certain high-risk results, such as abnormal results that are suspicious for cancer, require additional attention and management. For such high-risk tests, care coordination can help patients navigate healthcare processes, overcome barriers in obtaining care, and ensure appropriate follow-up has been received in response to the abnormal results.

This section will help you develop appropriate team structures to ensure patients receive appropriate follow up of test results.

ACTION STEPS

Step 1: Designate a care-coordinator to ensure completion of follow-up of specific high-risk tests vulnerable to follow-up failures

Change Approach:

Create a care coordinator or navigator position to assist patients in maneuvering the healthcare processes related to obtaining follow up of high-risk test results.

Provide care coordinators with tools to track follow up to completion.

D. Facilitate Time for Providers to Review and Act upon Test Results

To effectively review and respond to test results, providers must have sufficient dedicated time to consider and act on information. Lack of sufficient dedicated time for non-face-to-face patient care, such as managing test results, may force providers to rush through messages, leading to missing of important information. Additionally, insufficient time can lead to provider burnout, which can also reduce the quality and safety of care. Prior work on this topic suggest providers need approximately 1 hour per day for managing inbox messages, including test results. Additional time may be required for other non-face-to-face activities.

This section will help you determine the amount of time necessary for clinicians to process notifications and enable allocation of an appropriate amount of protected non-face time into their daily or weekly schedules.

ACTION STEPS

Step 1: Provide sufficient administrative time for non-face-to-face notification processing[29]

Change Approach:

Determine the amount of time (e.g., half-day per week, or 1 hour per day) necessary for non-face-to-face care activities by reviewing literature, interviewing providers, or performing time motion studies.

Integrate non-face-to-face care time directly into providers schedules.

© 2024 Zubkoff L et al. *JAMA Network Open*.

3. Implement Processes to Close the Loop on Test Results Reporting and Follow-Up

A. Ensure Providers and Staff Have Necessary Patient Contact Information for Fail-Safe Communication[29, 30]

This secondary driver pertains to the workflow of contacting a patient. Staff must obtain and maintain reliable ways to contact patients. The process of updating the patient's preferred method of communication is covered in Driver 1.A, "Increase Access to Test Results".

ACTION STEPS

Step 1: Ensure personnel involved in reporting test results have access to regularly updated contact information[30]

Change Approach:

Ensure that all providers and staff know where to find the standard patient contact information in the medical record.

Assess whether processes to collect and confirm patients' contact information at your site (e.g. who asks patients, who has the power to update, how often are detailed vs. basic questions asked) are reliable.

Develop a contingency plan for failures related to the patient's preferred form of communication (e.g. no reply to multiple voice mails, voice mail box full, patient is out of town for 2 months). Explore ways that patients' contact information may not fit into usual patterns (e.g. if patient has a mailing address only but no phone, if patient's listed mailing address is not their own or is a business rather than residential, if patient has no fixed address). Make your plan about accessing contact information fail-safe in these situations.

© 2024 Zubkoff L et al. *JAMA Network Open*.

3. Implement Processes to Close the Loop on Test Results Reporting and Follow-Up

B. Monitor for Breakdowns in Test Results Review and Communication[17, 30]

This section will help you prepare multiple processes that detect when patients have not received test results, or when appropriate follow-up actions have not been taken on tests.

ACTION STEPS

Step 1: Perform retrospective chart audits of follow-up on sample of patients with normal and abnormal results[17]

Change Approach:

Choose a time period of interest, a site of interest (for example, PACT Blue Clinic from April 8 – 12) and certain test results. Select a list of patients seen at that site/time who have those test results. Record how many audited charts have documented patient communication for selected normal and abnormal results.

Step 2: Use proactive strategies such as EHR based triggers to identify and intervene in potential delays in care[24]

Change Approach:

Use electronic trigger algorithms to identify patients whose abnormal results indicate need for follow-up action, and who are overdue for this action. Decide on a reasonable frequency at which to run this e-trigger (e.g., monthly). Please review the guide to implementing electronic triggers (InSTRuCt e-Trigger Manual)

Step 3: Support failsafe communication of abnormal results with patients.

Change Approach:

Develop policies and procedures that recommend direct (e.g., verbal) communication of high-risk abnormal results to patients. Allow providers to track whether patient portal results have been reviewed by patients.

Step 4: Use data for creating improvement strategies

Change Approach: Use data gathered in Steps 1–3 to determine which stage(s) in communication is/are most important for your site to improve. Take note of when you begin any improvement strategies, and track over time the impact of any changes you make to communication processes.

© 2024 Zubkoff L et al. *JAMA Network Open*.

3. Implement Processes to Close the Loop on Test Results Reporting and Follow-Up

C. Employ Standardized and Fail-Safe Processes and Policies for Communicating Abnormal Test Results to Providers[30]

This section will help you develop procedures for communication of test results among healthcare personnel (e.g. from lab or radiology to physician), and to evaluate any existing communication procedures.

ACTION STEPS

Step 1: Ensure all diagnostic services staff have a procedure in place for conveying results to the responsible provider[30]

Change Approach:

Ensure diagnostic services staff/managers have a communication plan for test results that considers exceptions to usual workflow. For example, what if the responsible provider is recorded incorrectly or has left the institution? (See page 16 and 18 of CTR Toolkit)

Facilities should maintain up-to-date contact information for providers and diagnostic services.

Step 2: Use standardized test results notification procedures that escalate to supervisory level when initial communication is not achieved[30]

Change Approach:

Identify supervisors for each clinic or site and develop a structured process that escalates results to supervisors if not acted on within a certain period of time. Example algorithms for certain critical results are available and can be useful.[30] (See page 16 and 28 of CTR Toolkit)

Step 3: Ensure that your site has an operational policy for results reporting. Check your policy for consistency with the "Eight Recommendations for Policies."[14]

Change Approach:

Draft and implement policies and procedures for communication of results from diagnostic services to providers. Include considerations such as:

- acceptable length of time between testing and reporting to the team[29],
- direct verbal communication of urgent or emergent critical results
- acceptable length of time between test result availability and notifying patients.[29]

© 2024 Zubkoff L et al. *JAMA Network Open*.

3. Implement Processes to Close the Loop on Test Results Reporting and Follow-Up

D. Use EHR Features to Support Closing the Loop on Test Results[31]

This section will help you use automation and electronic means to increase the rate of results that are communicated to patients, and the rate of clinical follow up on lab tests that require it.

ACTION STEPS

Step 1: Automatically make results available to patients via portal

Change Approach:

Enable CPRS / My HealtheVet to facilitate automated release of results to patients, e.g. as a My HealtheVet secure message. Sites may choose to apply this policy to different classes of lab results (e.g. release all normal labs, release all hemoglobin A1c results), or using differing timeframes (e.g., lab results released at 4 days, imaging results released at 1 week). Please work with your IT/Informatics team as and when needed.

Step 2: Use EHR features that enable coverage by a surrogate provider when out of office[23]

Change Approach:

Implement a coverage system for test results that return to a provider who is out of the office.

Ensure (at a system-wide level) provider awareness of the ability to designate a surrogate (a.k.a. delegate or covering provider) who will manage electronic laboratory alerts for a given provider if she/he is not available. Pay attention to less common provider types such as part-time, contract, or trainees.

Step 3: Use mandatory notification methods for high-priority abnormal and amended test results[29, 30, 32, 33]

Change Approach:

Work with IT to investigate current notification practices for high-priority abnormal results and amended results. Revise policies and procedures if needed, to ensure that the mandatory alerts are standardized and restricted down to the recommended list of core notification types and recommended mandatory notifications.[33]

See also Change Package section 2-A entitled “Prevent provider EHR notification fatigue.”

© 2024 Zubkoff L et al. *JAMA Network Open*.

CHANGE PACKAGE DEVELOPMENT:

The change package has been developed using a sociotechnical model[34] for understanding health information technology in complex healthcare systems (see Table 1). The global aim is to reduce missed test results. The InSTRuCt Driver Diagram (Figure 3) displays the relationship of this global aim to the primary drivers that contribute to achieving that aim and the subsequent action steps that are necessary to achieve the primary drivers. It clearly highlights the progression of steps an organization should take towards reduction of missed test results.

Table 1. Socio-technical Dimensions[17] Comprising the "Structure" of the Safer Dx Framework

Dimension	Description
Hardware and software	Computing infrastructure used to support and operate clinical applications and devices
Clinical content	The text, numeric data, and images that constitute the "language" of clinical applications
Human-computer interface	All aspects of technology that users can see, touch, or hear as they interact with it
People	Everyone involved with patient care and/or who interacts in some way with health care delivery (including those who manage health technology). This would include patients, clinicians and other health care personnel, IT developers and other IT personnel, and informaticians
Workflow and communication	Processes to ensure that patient care is carried out effectively
Internal organizational features	Policies, procedures, work environment, and culture
External rules and regulations	Federal or state rules that facilitate or constrain preceding dimensions
Measurement and monitoring	Processes to evaluate both intended and unintended consequences

© 2024 Zubkoff L et al. *JAMA Network Open*.

DEFINITIONS:

a. **Change Package.** A change package is a catalogue of strategies, change concepts, and action steps that guide participants in their improvement efforts.

(1) **Strategies**. Organizing framework for achieving systemwide improvements

(2) **Change concepts**. Approaches for specific changes

(3) **Action steps**. Specific steps for implementation of change concepts

b. **Driver Diagram.** A driver diagram is a visual depiction of a theory behind an improvement effort. It consists of Aims, Primary Drivers, and Secondary Drivers/Interventions.

(1) **Aim**. A clearly articulated goal or objective describing the desired outcome

(2) **Primary Driver**. System components or factors that contribute directly to achieving the aim.

(3) **Secondary Driver**. Action, interventions or lower-level components necessary to achieve the primary driver

c. **Closed-loop communication.** this involves acknowledging receipt of information and clarifying accuracy of that information with the message sender.

d. **Diagnostic Provider.** A diagnostic provider is a provider who performs or supervises the performance and interpretation of diagnostic tests either through privileges or by acting under a scope of practice.

e. **Ordering Provider.** An ordering provider is a provider authorized to enter and sign orders for diagnostic tests.

f. **Patient Notification.** Patient notification is communicating test results to patients or, if appropriate, to their personal representatives, including additional context and follow-up action as needed. Patient notification could occur through any synchronous or asynchronous method. For certain types of tests and certain types of patients, synchronous methods are preferred.

g. **Personal Representative.** A personal representative is a person, who under applicable law, has authority to act on behalf of the individual. This may include power of attorney, legal guardianship of an individual, the executor of the estate of a deceased individual, or someone under Federal, state, local or tribal law with such authority (e.g., parent of a minor).

h. **Supervising Practitioner.** Supervising practitioner refers to a licensed, independent practitioner, who has been credentialed and privileged at a VA medical facility in accordance with applicable requirements. ***NOTE:*** *'Supervising practitioners' are often referred to as 'attendings'. See VHA Handbooks 1400.01, Resident Supervision, and 1400.04, Supervision of Associated Health Trainees.*

i. **Synchronous Communication.** Synchronous communication is when parties involved in a communication are all present at the same time, such as in person, telephone conversations, or Clinical Video Telehealth (CVT).

© 2024 Zubkoff L et al. *JAMA Network Open*.

j. **Test Result.** Test results include the results of laboratory and pathology testing, diagnostic imaging, and diagnostic procedures. Test results are categorized as abnormal or normal as determined by a clinical provider and are further defined as follows:

(1) **Abnormal Test Results.** Abnormal test results are results that fall outside a specified normal reference range, are unexpected, or could indicate the presence of disease. An abnormal test may or may not require action and therapeutic intervention, depending on the clinical context. There are three types of abnormal test results that require action or therapeutic intervention:

(a) Critical Life Threatening. Any diagnostic finding which must be acted upon by the ordering provider or their designee immediately or within a short window of time and could result in severe morbidity or mortality if left untreated. (Example: critically elevated Potassium).

(b) Urgent Non-Life Threatening. Any diagnostic finding which must be acted upon by the ordering provider or their designee within a relatively urgent timeframe (as clinically indicated to ensure timely, appropriate and effective therapeutic action). An example of this is a Chest x-ray with newly discovered nodule, which is categorized as “Critical Not Life Threatening” with an Equivalent Radiology code such as 1001-Significant abnormality - attention needed or 1003-Possible malignancy.

(c) Clinically Significant. A diagnostic finding that requires action by the ordering provider, or their designee, but not necessarily in an immediate or urgent time-frame. (Example: High Cholesterol).

(2) **Normal Test Results.** While the significance of a “normal” test result needs to be determined clinically, in the context of this Directive it is defined as a diagnostic finding that falls within the normal reference range for the test and may or may not require immediate action or change in treatment depending on clinical circumstances. (Example: Patient on Warfarin whose international normalized ratio (INR) is sub-therapeutic but in the “normal” range; low-density lipoprotein (LDL) =120mg/dl with coronary disease) Radiologic tests can also be considered normal when radiologists use something equivalent “*Radiology Code: 1000-No Alert Required-No significant finding or provider is already aware.”*

© 2024 Zubkoff L et al. *JAMA Network Open*.

REFERENCES:

1. Dahm MR, Georgiou A, Herkes R, Brown A, Li J, Lindeman R, et al. Patient groups, clinicians and healthcare professionals agree - all test results need to be seen, understood and followed up. Diagnosis (Berl). 2018;5(4):215-22.
2. Ammenwerth E, Schnell-Inderst P, Hoerbst A. The impact of electronic patient portals on patient care: a systematic review of controlled trials. J Med Internet Res. 2012;14(6):e162.
3. Christensen K, Sue VM. Viewing Laboratory Test Results Online: Patients' Actions and Reactions. Journal of Participatory Medicine. 2013;5.
4. Donabedian A. The quality of care. How can it be assessed? JAMA: The Journal of the American Medical Association. 1988;260(12):1743-8.
5. Pillemer F, Price RA, Paone S, Martich GD, Albert S, Haidari L, et al. Direct Release of Test Results to Patients Increases Patient Engagement and Utilization of Care. PLoS One. 2016;11(6):1-9.
6. Zhong X, Liang M, Sanchez R, Yu M, Budd PR, Sprague JL, et al. On the effect of electronic patient portal on primary care utilization and appointment adherence. BMC Med Inform Decis Mak. 2018;18(1):84.
7. Schwappach DL. Review: engaging patients as vigilant partners in safety: a systematic review. Med. Care Res. Rev. 2010;67(2):119-48.
8. Griffin A, Skinner A, Thornhill J, Weinberger M. Patient Portals: Who uses them? What features do they use? And do they reduce hospital readmissions? Appl Clin Inform. 2016;7(2):489-501.
9. Oest SER, Hightower M, Krasowski MD. Activation and Utilization of an Electronic Health Record Patient Portal at an Academic Medical Center-Impact of Patient Demographics and Geographic Location. Acad Pathol. 2018;5:2374289518797573.
10. Krasowski MD, Grieme CV, Cassady B, Dreyer NR, Wanat KA, Hightower M, et al. Variation in Results Release and Patient Portal Access to Diagnostic Test Results at an Academic Medical Center. J Pathol Inform. 2017;8:45.
11. Davis RE, Koutantji M, Vincent CA. How willing are patients to question healthcare staff on issues related to the quality and safety of their healthcare? An exploratory study. Qual. Saf Health Care. 2008;17(2):90- 6.
12. Davis RE, Jacklin R, Sevdalis N, Vincent CA. Patient involvement in patient safety: what factors influence patient participation and engagement? Health Expect. 2007;10(3):259-67.
13. Singh H, Thomas EJ, Mani S, Sittig DF, Arora H, Espadas D, et al. Timely follow-up of abnormal diagnostic imaging test results in an outpatient setting: are electronic medical records achieving their potential? Arch Intern Med. 2009;169(17):1578-86.
14. Giardina TD, Baldwin JL, Sittig DF, Singh H. Patient Perceptions of Test Result Notification Via the Patient Portal. 2016 Public Health Informatics Conference. 2016.
15. Giardina TD, Modi V, Parrish D, Singh H. The Patient Portal and Abnormal Test Results: An Exploratory Study of Patient Experiences. Patient Experience Journal. 2015;2(1):Article 20.
16. Johnson AJ, Easterling D, Nelson R, Chen MY, Frankel RM. Access to radiologic reports via a patient portal: clinical simulations to investigate patient preferences. J. Am. Coll. Radiol. 2012;9(4):256-63.
17. Eder M 2013;Pageshttps://www.ahrq.gov/sites/default/files/wysiwyg/professionals/quality-patient-safety/quality-resources/tools/lab-testiing/lab-testing-toolkit.pdf.
18. Veterans Health Administration. Communication of Test Results Toolkit. 2012.
19. Zikmund-Fisher BJ, Exe NL, Witteman HO. Numeracy and literacy independently predict patients' ability to identify out-of-range test results. J. Med. Internet. Res. 2014;16(8):e187.
20. Zikmund-Fisher BJ, Scherer AM, Witteman HO, Solomon JB, Exe NL, Tarini BA, et al. Graphics help patients distinguish between urgent and non-urgent deviations in laboratory test results. J Am Med Inform Assoc. 2017;24(3):520-8.
21. Shah T, Patel-Teague S, Kroupa L, Meyer AND, Singh H. Impact of a national QI programme on reducing electronic health record notifications to clinicians. BMJ Qual Saf. 2019;28(1):10-4.
22. Sittig DF, Ash JS, Singh H. The SAFER guides: empowering organizations to improve the safety and effectiveness of electronic health records. Am J Manag Care. 2014;20(5):418-23.

© 2024 Zubkoff L et al. *JAMA Network Open*.

23. Singh H, Wilson L, Reis B, Sawhney MK, Espadas D, Sittig DF. Ten Strategies to Improve Management of Abnormal Test Result Alerts in the Electronic Health Record. Journal of Patient Safety. 2010;6(2):121-3.
24. Meyer AN, Murphy DR, Singh H. Communicating Findings of Delayed Diagnostic Evaluation to Primary Care Providers. J Am Board Fam. Med. 2016;29(4):469-73.
25. Murphy DR, Satterly T, Giardina TD, Sittig DF, Singh H. Practicing Clinicians' Recommendations to Reduce Burden from the Electronic Health Record Inbox: a Mixed-Methods Study. J Gen Intern Med. 2019.
26. Murphy DR, Satterly T, Rogith D, Sittig DF, H. S. Barriers and Facilitators Impacting Reliability of the Electronic Health Record-Facilitated Total Testing Process. International Journal of Medical Informatics. 2019.
27. Murphy DR, Giardina TD, Satterly T, Sittig DF, Singh H. An Exploration of Barriers, Facilitators, and Suggestions for Improving Electronic Health Record Inbox-Related Usability: A Qualitative Analysis. JAMA Netw Open. 2019;2(10):e1912638.
28. Langley GL, Moen R, Nolan KM, Norman CL, Provost LP. The Improvement Guide: A Practical Approach to Enhancing Organizational Performance. Second Edition ed. San Francisco: Jossey-Bass Publishers; 2009.
29. VHA Directive 1088: Communicating Test Results to Providers and Patients. Washington DC. ; 2015.
30. Singh H, Vij MS. Eight recommendations for policies for communicating abnormal test results. Jt Comm J Qual Patient Saf. 2010;36(5):226-32.
31. Smith M, Murphy D, Laxmisan A, Sittig DF, Reis B, Esquivel A, et al. Developing software to "track and catch" missed follow-up of abnormal test results in a complex sociotechnical environment. Appl. Clin. Inform. 2013;4(3):359-75.
32. Singh H, Thomas EJ, Sittig DF, Wilson L, Espadas D, Khan MM, et al. Notification of abnormal lab test results in an electronic medical record: do any safety concerns remain? Am. J. Med. 2010;123(3):238-44.
33. Singh H, Sittig DF. Advancing the science of measurement of diagnostic errors in healthcare: the Safer Dx framework. BMJ Qual Saf. 2015;24(2):103-10.
34. Sittig DF, Singh H. A new sociotechnical model for studying health information technology in complex adaptive healthcare systems. Qual Saf Health Care. 2010;19(Suppl 3):i68-i74.

© 2024 Zubkoff L et al. *JAMA Network Open*.

ACKNOWLEDGEMENTS:

The following individuals and organizations were vital in the development of this change package:

Organizations:

- Veterans Affair's Health Services Research and Development Service (HSR&D)
- Michael E. DeBakey VA Medical Center
- Baylor College of Medicine

Expert Panel:

- Peter D. Mills PhD, MS *Director at **VHA National Center for Patient Safety Field Office***
- Julia B. Neily *Associate Director at **VHA National Center for Patient Safety Field Office***
- Thomas H. Payne, MD, FACP *Medical Direct, IT Services, **UW Medicine***
- Molly Horstman, MD, MS A*ssistant Professor at* **Baylor College of Medicine**
- Conrad Schuerch, MD *Chairman Emeritus, Laborator Medicine* ***Geisinger***
- Melissa Yu, MD, FAAN *Associate Professor, **Baylor College of Medicine***
- Gilad Kuperman, PhD *Associate Chief Health Informatics Officer at **Memorial Sloan Kettering Cancer Center***
- Himabindu Kadiyala, MD *Internal Medicine **Michael E DeBakey VA Medical Center***

Project Team:

- Hardeep Singh, MD, MPH, *Professor of Medicine*
- Daniel R. Murphy, MD, MBA, *Instructor*
- Andrew J. Zimolzak, MD, *Physician*
- Traber Giardina, PhD, *Assistant Professor*
- Lisa Zubkoff, PhD, *Assistant Professor of Psychiatry*
- Umber Shahid, PhD, *Research Associate*
- Umair Mushtaq, MS, *Research Coordinator*
- Sahar Memon, MPH, MS *Health Science Specialist*

© 2024 Zubkoff L et al. *JAMA Network Open*.

eTable 2. Detailed Coding of Interventions Implemented by Site

**Primary Drivers**	**Patient Engagement**			**Situational Awareness**				**Closing the Loop**				**Other Interventions**							
Secondary Drivers	Increase access to test results	Educate patients on expectations of test result and follow-up process	Increase patient comprehension of test results	Prevent provider EHR notification fatigue	Ensure effective teamwork in management of test results	Create a support structure to facilitate test results review and follow-up	Facilitate time for providers to review and act upon test results	Ensure providers and staff have necessary contact information for fail safe communication	Monitor for breakdowns in test results review and communication	Employ standardization and failsafe processes and policies for communicating abnormal test results	Use EHR features to support closing the loop on test results	Update polices and procedures	Education of providers other than alert fatigue	Increase (measure) communication of MISSED test results to patients	Demonstrated use of QI tools (e.g., process map, pdsa cycles)	Procedure to more effectively label an abnormal test	Pay for performance	Transportation	**Total Number of Interventions Implemented by Site**
Site 1	1	0	0	1	0	0	0	0	0	0	0	1	0	0	0	0	0	0	***3***
Site 2	1	0	0	0	0	0	0	0	1	0	0	0	0	0	1	0	0	0	***3***
Site 3	1	0	1	1	1	1	0	0	1	0	0	0	1	0	0	1	0	0	***8***
Site 4	1	0	0	1	0	0	0	0	0	0	0	0	0	0	0	1	0	0	***3***
Site 5	1	0	0	1	0	0	0	0	0	0	1	0	0	0	0	0	0	0	***3***
Site 6	0	1	1	0	1	0	0	1	0	0	1	0	0	0	1	0	0	0	***6***
Site 7	1	1	0	0	0	0	0	0	1	0	0	0	0	0	0	0	0	0	***3***
Site 8	1	0	0	0	1	0	0	0	1	0	0	0	0	0	0	0	0	0	***3***
Site 9	1	0	0	1	0	0	0	0	1	0	1	0	0	0	0	0	0	0	***4***
Site 10	1	1	0	0	1	0	0	0	0	1	0	0	0	0	0	0	1	0	***5***
Site 11	1	0	0	1	0	0	0	1	0	0	1	1	0	0	1	0	0	1	***7***
**Total Sites Using Driver**	***10***<br>***91%***	***3***<br>***27%***	***2***<br>***18%***	***6***<br>***55%***	***4***<br>***36%***	***1***<br>***9%***	***0***<br>***0%***	***2***<br>***18%***	***5***<br>***45%***	***1***<br>***9%***	***4***<br>***36%***	***2***<br>***18%***	***1***<br>***9%***	***0***<br>***0%***	***3***<br>***27%***	***2***<br>***18%***	***1***<br>***9%***	***1***<br>***9%***	

© 2024 Zubkoff L et al. *JAMA Network Open*.

eFigure 2. Percentage of follow-up abnormal test results for colorectal cancer e-trigger across the three study phases. Each point represents the follow-up rate for one calendar month of data from one site. Data from all 3 cohorts are presented.

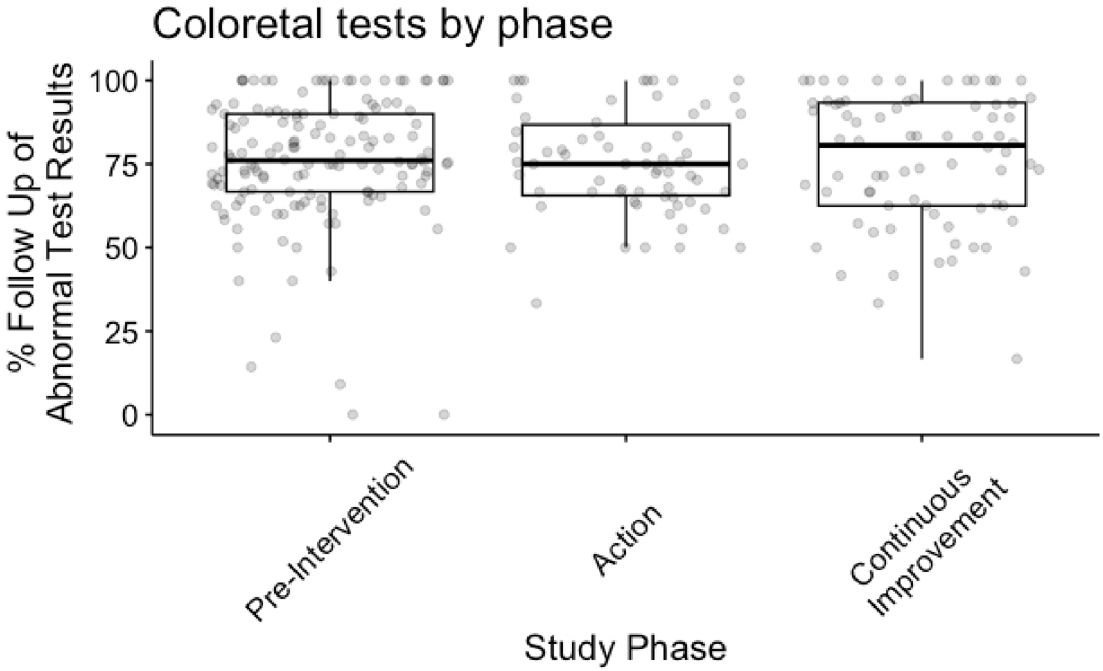

© 2024 Zubkoff L et al. *JAMA Network Open*.

eFigure 3. Percentage of follow-up abnormal test results for lung cancer e-trigger across the three study phases. Each point represents the follow-up rate for one calendar month of data from one site. Data from all 3 cohorts are presented.

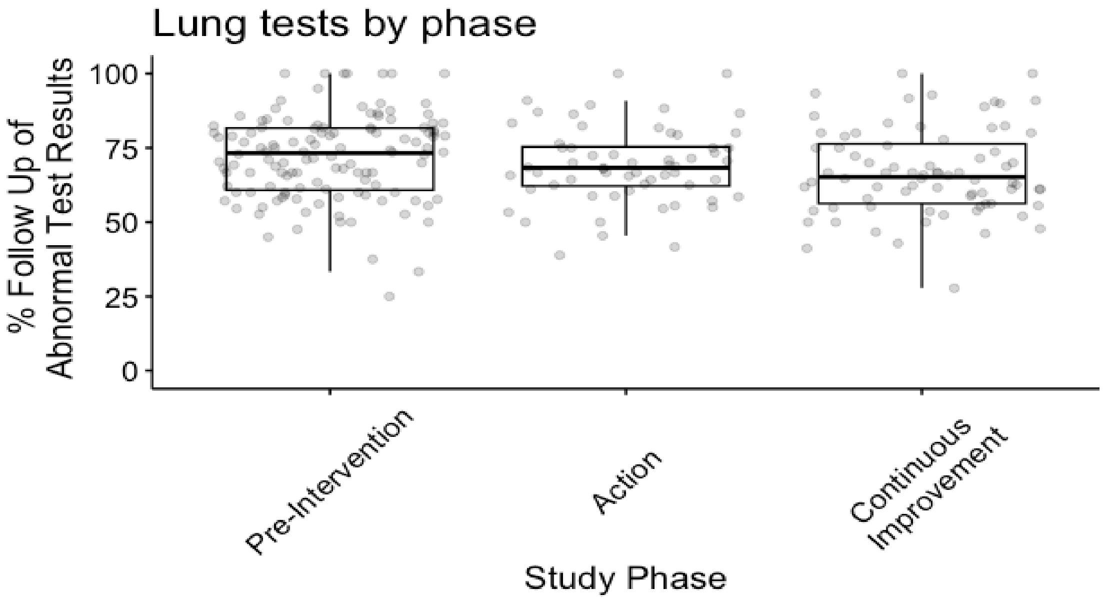

© 2024 Zubkoff L et al. *JAMA Network Open*.

eFigure 4. Results of the intervention across cohorts. We conducted ANOVAs for each cohort. For the colorectal data, we found no effect of study phase for any of the three cohorts (p>0.05 for all).

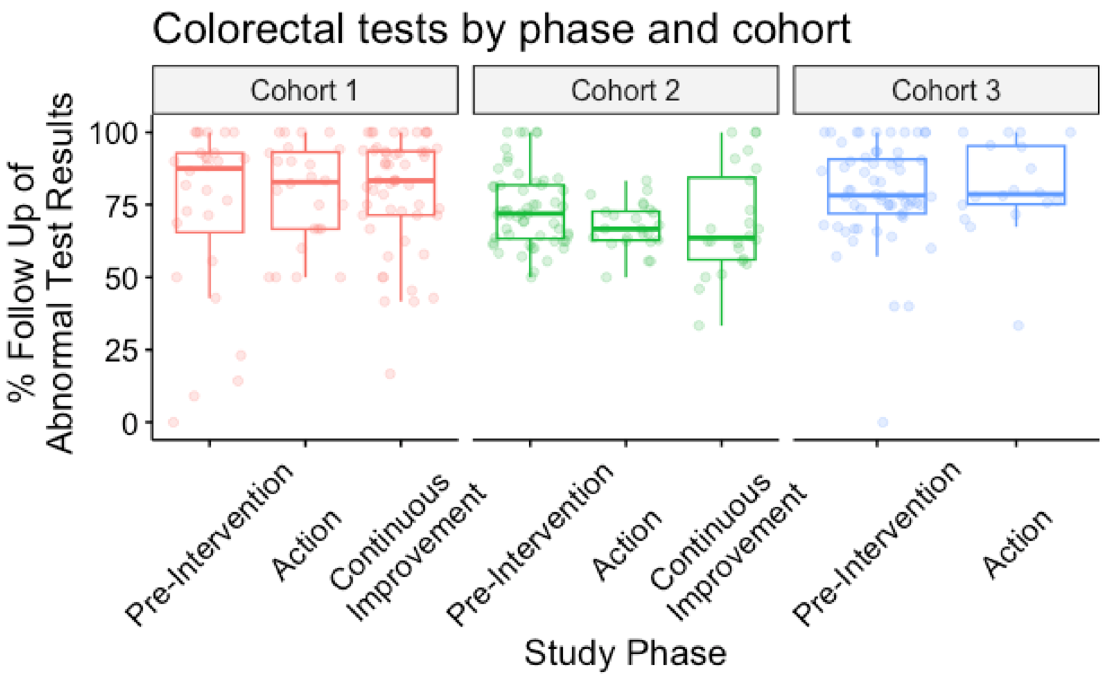

© 2024 Zubkoff L et al. *JAMA Network Open*.

eFigure 5. Results of the intervention across cohorts. We conducted ANOVAs for each cohort. For the lung data, there were no significant effects of study phase for Cohorts 1 or 3 ($p>0.05$ for both), but we did find a significant effect of study phase for cohort 2 ($F=5.55$, $p=0.005$). By inspection, lung e-trigger follow-up performance decreased in each phase for Cohort 2 (green points and boxes in). Specifically, the mean follow-up rate was 72.3% in pre-intervention, 66.3% in action phase, and 60.8% in continuous improvement.

Cohort 1 was the group most interrupted by the start of the COVID pandemic, so we hypothesized that they might see a null effect of the intervention, whereas other cohorts would see a positive effect. Altogether, these data show no significant impact of the pandemic because null effects are seen in at least one e-trigger across all 3 cohorts.

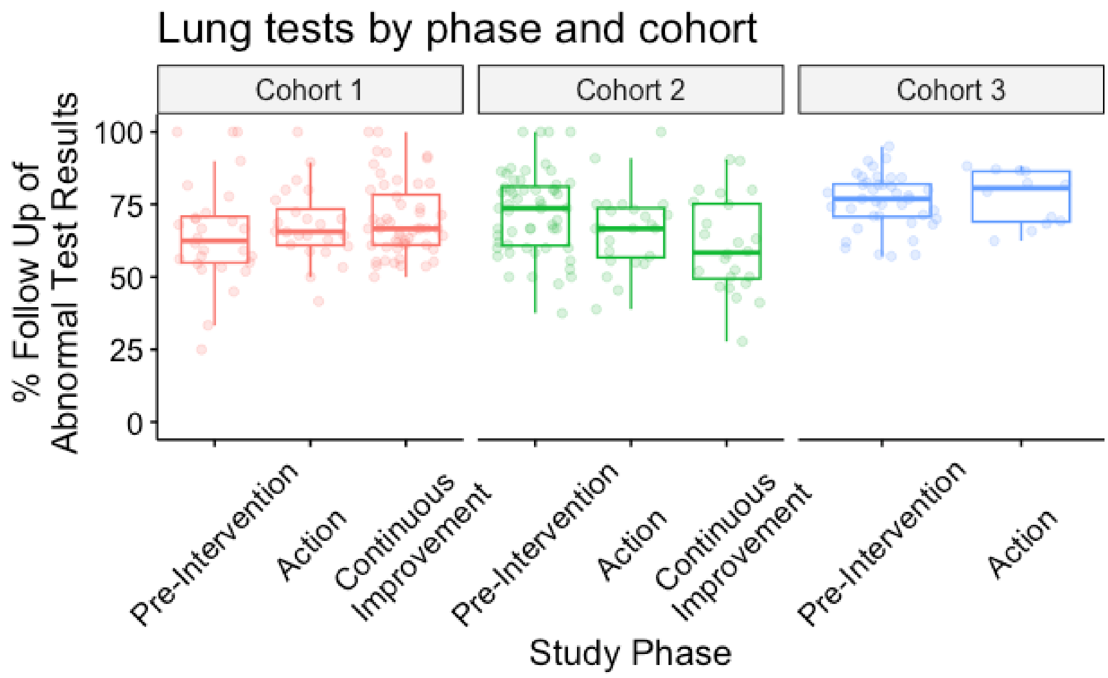

© 2024 Zubkoff L et al. *JAMA Network Open*.
