# Supplementary material for: A Virtual Breakthrough Series Collaborative for Missed Test Results: A Stepped-Wedge Cluster-Randomized Clinical Trial
Source: JAMA Netw Open. 2024 Oct 30;7(10):e2440269. doi: 10.1001/jamanetworkopen.2024.40269 (PMC11525607; doi:10.1001/jamanetworkopen.2024.40269)
Supplement: Supplement 3. — Data Sharing Statement [file jamanetwopen-e2440269-s003.pdf]

Data Sharing Statement

Zubkoff. A Virtual Breakthrough Series Collaborative for Missed Test Results. *JAMA Netw Open*. Published October 30, 2024. doi:10.1001/jamanetworkopen.2024.40269

Data
Additional Information: Clinicaltrials.gov: NCT04166240
Data available: No
